# Genomic insights into the evolutionary history and diversification of bulb traits in garlic

**DOI:** 10.1186/s13059-022-02756-1

**Published:** 2022-09-07

**Authors:** Ningyang Li, Xueyu Zhang, Xiudong Sun, Siyuan Zhu, Yi Cheng, Meng Liu, Song Gao, Jiangjiang Zhang, Yanzhou Wang, Xiai Yang, Jianrong Chen, Fu Li, Qiaoyun He, Zheng Zeng, Xiaoge Yuan, Zhiman Zhou, Longchuan Ma, Taotao Wang, Xiang Li, Hanqiang Liu, Yupeng Pan, Mengyan Zhou, Chunsheng Gao, Gang Zhou, Zhenlin Han, Shiqi Liu, Jianguang Su, Zhihui Cheng, Shilin Tian, Touming Liu

**Affiliations:** 1 Institute of Bast Fiber Crops, Chinese Academy of Agricultural Sciences, Changsha, 410205 China; 2 Shandong Agricultural University, Tai’an, 271018 China; 3 Novogene Bioinformatics Institute, Beijing, 100083 China; 4 Yangzhou University, Yangzhou, 225009 China; 5 Industrial Research Institute of garlic (IBFC-Jinxiang), Jinxiang, 272200 China; 6 Changsha University, Changsha, 410003 China; 7 Shandong Dongyun Research Center of garlic Engineering, JinXiang, 272200 China; 8 Northwest A&F University, Yangling, 712100 China; 9 University of Hawaii at Manoa, Honolulu, 96822 USA

## Abstract

**Background:**

Garlic is an entirely sterile crop with important value as a vegetable, condiment, and medicine. However, the evolutionary history of garlic remains largely unknown.

**Results:**

Here we report a comprehensive map of garlic genomic variation, consisting of amazingly 129.4 million variations. Evolutionary analysis indicates that the garlic population diverged at least 100,000 years ago, and the two groups cultivated in China were domesticated from two independent routes. Consequently, 15.0 and 17.5% of genes underwent an expression change in two cultivated groups, causing a reshaping of their transcriptomic architecture. Furthermore, we find independent domestication leads to few overlaps of deleterious substitutions in these two groups due to separate accumulation and selection-based removal. By analysis of selective sweeps, genome-wide trait associations and associated transcriptomic analysis, we uncover differential selections for the bulb traits in these two garlic groups during their domestication.

**Conclusions:**

This study provides valuable resources for garlic genomics-based breeding, and comprehensive insights into the evolutionary history of this clonal-propagated crop.

**Supplementary Information:**

The online version contains supplementary material available at 10.1186/s13059-022-02756-1.

## Background

Garlic (*Allium sativum*) contains rich compound of S-alk(en)yl-L-cysteine sulfoxides that confer a unique flavor and medicinal effect and is grown worldwide in all temperate to subtropical area as an important vegetable, spice, and medicinal crop, with an annual production of approximately 28.2 million tons (Data from FAO; http://faostat.fao.org/). Garlic has been cultivated and widely used since prehistoric times when the historical traces fade away and cannot be followed either to a wild ancestor or even to the exact area of domestication [1]. Currently, garlic is proposed to originate in some regions of the Mediterranean and Central and West Asian, and to be domesticated from the wild *Allium longicuspis* [2]. However, the wild species *Allium tuncelianum* from Turkish showed a remarkable similarity to garlic and is considered as another candidate for the wild ancestor of garlic [3]. Unlike the case of the seed-bearing crops, garlic sterility has led to a much more restricted morphological and genetic variation in garlic, irrespective of the large area where it is in cultivation. Although genetic diversity in garlic has been frequently studied using morphological, physiological, isozyme, and molecular makers [2, 4–7], an exact population structure remains largely unknown, which limits the insights into evolutionary history of this *Allium* crop. Currently, the proposal about the infraspecific classification of garlic with four major groups and one subgroup is widely accept, in which the longicuspis group is presumably the basal group in the origination of cultivated garlic [7].

Comprehensive genomic variation maps are a powerful tool for the genetic diversity analysis and have been widely used for exploring the evolutionary history in scores of crops [8–10]. Although garlic is diploid species with only eight chromosome pairs, garlic nuclear genome is dramatically complex, with a huge size (16.9 Gb) and large ratio of repetitive sequences (91.3%). A recent study sequenced the garlic genome and addressed these challenges comprehensively with a repertoire of five advanced sequencing methodologies leading them to an assembly with a high degree of completeness [11], which provides a basis for detecting the genomic variations and exploring its evolutionary history. In this study, we first sequenced 230 garlic accessions to infer the population structure by genotype-by-sequencing (GBS) technology, and then explored the evolutionary history by genome-wide resequencing 84 accessions from three main garlic groups.

## Results

### Population structure

**Fig. 1:**
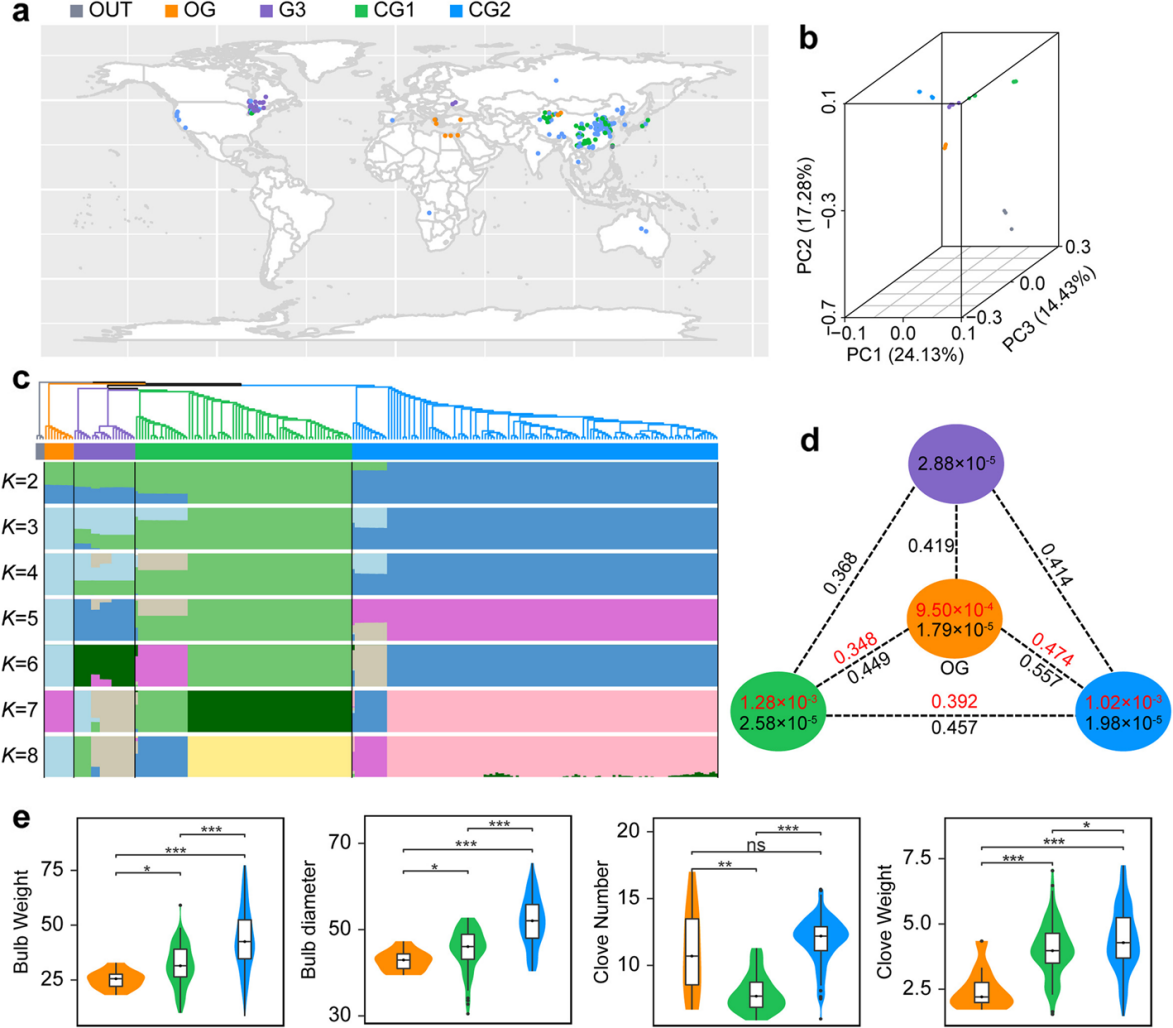
Geographic distribution, population structure, and genetic diversity of garlic accessions. **a** Geographic distribution of 230 garlic accessions, which are represented by dots on the world map. **b** Principal components (PC) analysis plots of the first three components of 230 accessions. **c** Phylogenetic tree and model-based clustering analysis of 230 accessions. The rooted tree constructed using neighbor-joining method. Model-based clustering analysis with different cluster numbers (*k* = 2–8). The *y*-axis quantifies cluster membership, and the *x*-axis lists the different accessions. The orders and positions of these accessions on the *x*-axis are consistent with those presented in the neighbor-joining tree. Three *Allium ampeloprosum* accessions were used as the outgroup species. **d** Nucleotide diversity (*π*) and population divergence (*F*_ST_) across the four groups. The value in each circle represents an estimation of nucleotide diversity for each group, and values on each line indicate pairwise population divergence between groups. Value in black or red indicates that it was estimated using the SNPs from genotyping-by-sequencing and whole-genome resequencing. **e** Comparison of the trait value in the origin group (OG) and two cultivated groups of East Asia, CG1 and CG2 for four bulb traits. ns indicates no significant difference, and *, **, *** indicates the significance at the level of 0.05, 0.01, and 0.001, respectively

A total of 233 diverse germplasms collected from 17 countries, including 230 garlic accessions and addition three accessions of *Allium ampeloprosum* that is a close related species of garlic [12], were used in this study (Fig. 1a, Additional file 1: Table S1). Sequencing of these 233 accessions using the genotyping-by-sequencing (GBS) technology generated a total of 2,036,304 SNPs (Additional file 1: Table S2). Interestingly, average 84.3% reads from *Allium ampeloprosum* accessions could be aligned into the garlic genome, covering 4.9% garlic genome, with a ~15.6-fold coverage depth, which indicated that there was a close phylogenetic relationship between garlic and *Allium ampeloprosum*.

We then inferred the population structure by the neighbor joining (NJ) tree, Bayesian clustering, and principal component analyses (PCA) using these SNPs, and three accessions of *Allium ampeloprosum* were used as the outgroup species. Our result indicated four main groups in the garlic population (Fig. 1b, c). Ten garlic accessions together with three *Allium ampeloprosum* accessions were located into a monophyletic clade at the root of tree, and most accessions were collected from the Central Asian regions which were deemed to be the origin center of garlic [7], suggesting that these ten accessions were nearer to wild species in the phylogenetic relationship than the others, and this group was designated as origin group (OG). Two main groups, most of which were the cultivars collected from China, were identified as two monophyletic clades, consisting of 74 and 125 accessions, and were defined as cultivated group 1 (CG1) and cultivated group 2 (CG2). Additionally, 21 accessions were assigned into the remaining G3 group (Fig. 1c), and all of them were the cultivars collected from Canada and Ukraine.

### Genomic variation map

Two main cultivated groups of China, CG1 and CG2, were focused on for further analysis. We explored the variations pattern in garlic genome by resequencing 84 core accessions, including all 10 OG accessions, 35 and 39 accessions from CG1 and CG2. A total of 13.51 Tb sequencing data was yielded and mapped to the garlic reference genome (PRJNA606385 in NCBI [13]), resulting in an average alignment rate of 99.05% with an average depth of 9.36-fold (Additional file 1: Table S3). Approximately 99.24% of mapped reads showed a match length of ≥ 100 bp (Additional file 2: Fig. S1). We identified 129,409,768 variants in garlic genome, including 120,857,927 SNPs and 8,551,841 small insertions or deletions (indels; ≤5 bp), with an average of 8.0 variants per kilobase in genome (Additional file 1: Tables S4 and S5; Additional file 2: Fig. S2). There were 3,102,210 SNPs (2.57%) and 224,019 (2.62%) indels located in the coding regions, of which 211,213 nonsynonymous SNPs and 7083 frameshift indels led potentially to important changes in protein sequences. These variants represent the first genome-wide variations on a large scale in garlic, and it provides a valuable resource for the studies of biology and breeding in this economically important crop.

### Population diversity and differentiation

To investigate the genetic diversity of population, we estimated the nucleotide diversity of 230 GBS-sequenced accessions and revealed a relatively low genetic diversity in four identified subpopulations, ranging from 1.79 × 10^−5^ to 2.88 × 10^−5^ (Fig. 1d). Investigation of genomic divergency among four subpopulations by estimating the pairwise genome-wide fixation index (*F*_ST_) value revealed a high *F*_ST_ value among them (>0.368; Fig. 1d). We also estimated the nucleotide diversity and genomic divergency in 84 resequenced accessions based on the genome-wide SNPs, and identified a nucleotide diversity of 9.50 × 10^−4^ (OG), 1.28 × 10^−3^ (CG1), 1.02 × 10^−3^ (CG2), respectively. Although the *F*_ST_ value observed in 84 accessions was slightly smaller than those estimated by GBS SNPs, it remained more than 0.348 (Fig. 1d), especially that between OG and CG2 (0.474), which was similar to that of two rice subspecies, *indica* and *japonica* (0.55) [14]. This result indicated a large genomic divergence among the groups of OG, CG1, and CG2. Additionally, dramatically large diversity of bulb traits was observed in garlic population. For example, among 230 accessions, the variation range of clove weight was from 0.57 to 7.27 g, with an average weight of 4.09 g, whereas the clove number ranged from 5.0 to 17.0, with average 10.4 cloves (Additional file 1: Table S1). Notably, significant difference of bulb traits could be observed among three garlic groups (Fig. 1e; Additional file 2: Fig. S3). That is, average 8.14 cloves were produced by accessions of CG1, while this number in OG and CG2 reached 11.05 and 11.88, respectively; for the clove weight, significant increase was observed in both two cultivated groups (~4.03 and ~4.40 g, respectively) comparing with the OG group (~2.49 g).

### Independent domestication of cultivated CG1 and CG2

**Fig. 2:**
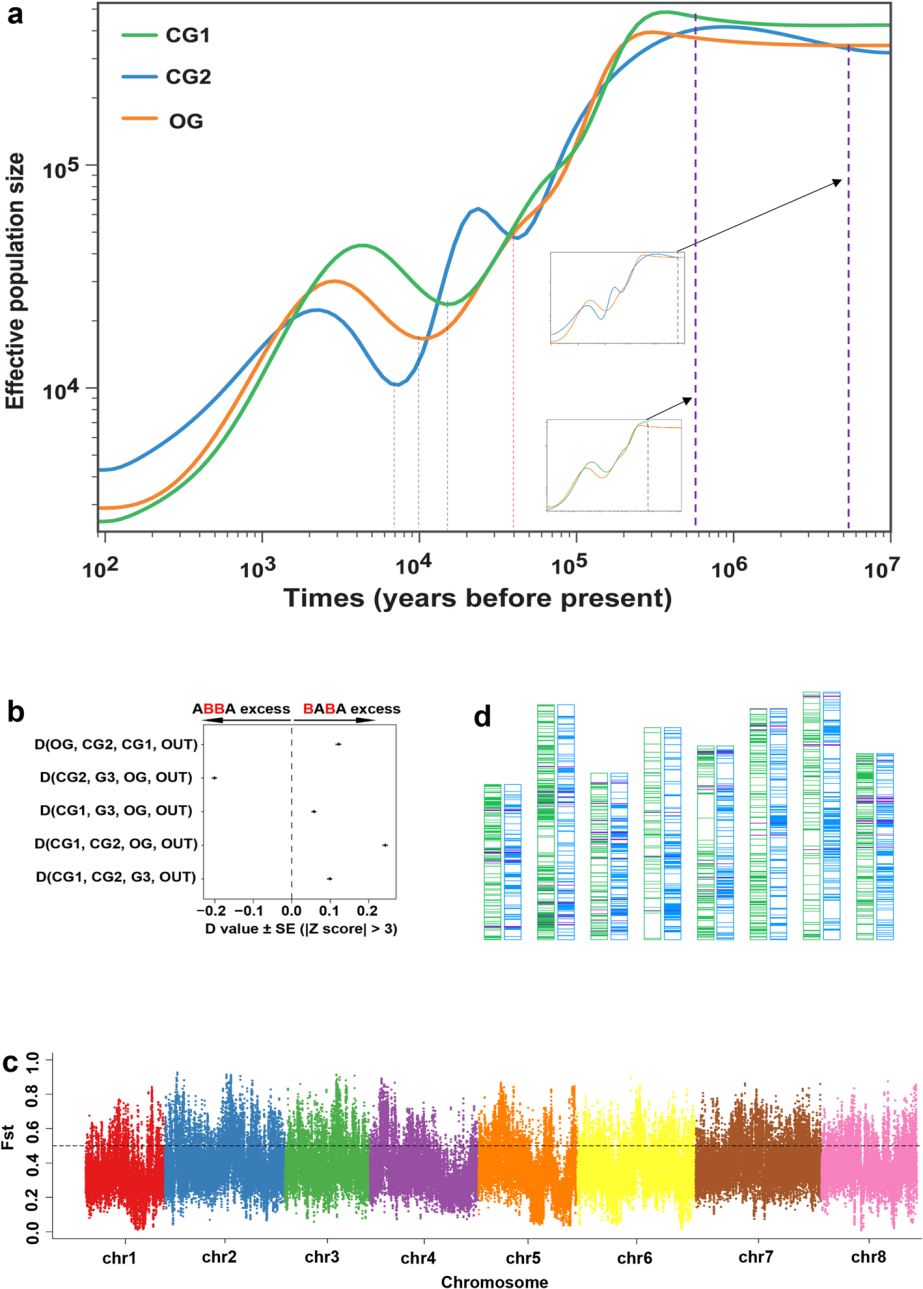
Evidence for the independent domestication of two cultivated groups of East Asia. **a** Demographic history of OG, CG1, and CG2 by estimating the historical effective population size *Ne*. Two dashed purple lines indicate the diverged time of CG1 and CG2 from OG. The dashed red line represents a time of the CG1 population expansion, whereas the dashed black lines indicate the bottleneck of three garlic groups. **b** Patterson’s D test for four garlic populations identified five introgression patterns with significant *D* values (|Z-score| > 3), using OUT as an outgroup. The introgression patterns from top to bottom in the chart indicated gene flows between OG and CG1, G3 and OG, CG1 and OG, CG1 and OG, and CG1 and G3, respectively. **c** The distribution of fixation index (*F*_ST_) of CG1 and CG2. A total of 4.89 Gb genome (30.1% of garlic genome) shows distinct divergency (*F*_ST_ > 0.5). **d** The distribution of putative selective sweeps in the genome. The order of chromosome from left to right is from chromosome 1 to chromosome 8 successively. For each pair chromosome, the left and right one shows the selective sweeps of CG1 and CG2 in green and blue lines, respectively. The purple lines represent the overlap of selective sweeps between CG1 and CG2

We explored the demographic history by estimating historical effective population size (*N*e), revealing that three garlic groups diverged in at least 500,000 years before present (YBP; Fig. 2a). Furthermore, the CG2 population rapidly expanded in ~40,000 YBP, and then, three populations underwent one demographic bottleneck in ~10,000–15,000 YBP, which coincided with the Younger Dryas event, a millennial-scale cold snaps of glacial time (~12,000 years ago) [15]. The demographic bottleneck around 10,000–15,000 YBP was further observed in the estimation of four populations, including these three whole-genome resequenced populations and G3, using GBS SNPs (Additional file 2: Fig. S4). Interestingly, we observed three populations which showed different sizes in their bottleneck period, and the populational scale increased from three different periods, i.e., CG1 expanded earlier than OG, whereas CG2 size increased in ~7000 YBP, which was later than that of OG (Fig. 2a). This analysis indicated a different evolutionary history among three garlic subpopulations, and it provided an evidence for supporting the conclusion that three garlic subpopulations diverged prior to the Younger Dryas event. Humans have a history of crop domestication with ~10,000 years [16], and the records about garlic in Sumerian and Akkadian cuneiform texts date back approximately to 2600 BC [17], and the earliest plant remains from Egypt were discovered in the tomb of Tutankhamun from 1337 BC [18]. Thus, our result suggested that the split of two cultivated groups, CG1 and CG2, was prior to the domestication of garlic. Cultivated garlic is sterile, which causes a reproductive isolation among garlic individuals; therefore, we proposed that, after CG1 and CG2 splitting as two groups, they were domesticated from two independent routes. Additionally, we have not observed gene flows between CG1 and CG2 from the estimation of D-statistic [19] based on the GBS SNPs (Fig. 2b), which further supported this proposal about the independent domestication event.

We observed that the genome of CG1 and CG2 showed distinct divergency, and 30.1% of which (4.89 Gb in total length) had an *F*_ST_ value of more than 0.5 (Fig. 2c, Additional file 1: Table S6). To better understand the domestication of CG1 and CG2, we analyzed the selective signal of two cultivated groups during garlic domestication based on three methods, namely the population branch statistic (PBS) approach, the estimation of the *θπ* ratio (*π*_OG_/*π*_CG_), and the test of cross-population composite likelihood ratio (XP-CLR; CG versus OG), and the genomic regions estimated with the top 5% value by at least two of three methods were considered as the putative selective sweeps. Finally, a total of 842 and 943 putative selective sweeps associated with domestication in CG1 and CG2 was identified, respectively, covering 3.50% and 5.26% of the garlic genome (567.9 and 854.8 Mb in total length) and comprising 1616 and 2775 garlic genes, respectively (Additional file 2: Fig. S5, Additional file 1: Tables S7-S10). Notably, only 0.18% of garlic genome (29.3 Mb) underwent selection commonly in CG1 and CG2 (Fig. 2d), thereby causing only 105 out of 57,561 garlic genes were shared in the sweeps of two cultivated groups (Additional file 1: Table S11), which is even less than that of *melo* and *agrestis*, two independently domesticated melon subspecies [9]. Therefore, these analyses provided further evidences for the independent domestication of CG1 and CG2.

### Distinct difference between the transcriptome of CG1 and CG2

**Fig. 3:**
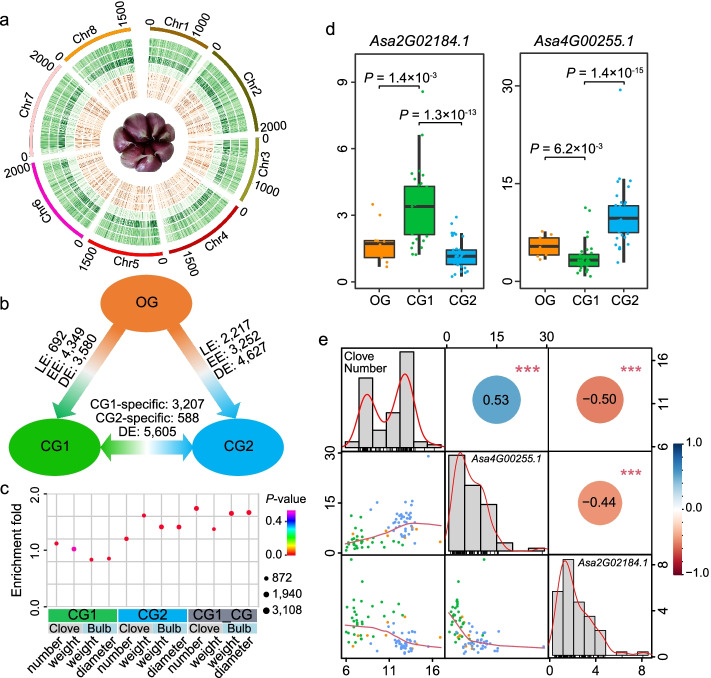
Expression evolution of garlic genes. **a** Feature of gene expression in three examined garlic groups. The tracks from inside to outside represent the FPKM value of gene in OG, CG1, and CG2, and Shannon-Wiener index of transcript abundance in OG, CG1, CG2, and in all 81 investigated accessions. **b** Gene number with change of expression among OG, CG1, and CG2. LE and EE indicate the number of gene that lose expression and emerges expression during the domestication history. DE represents the number of genes that express in both groups but have significant expression difference. **c** Enrichment analysis of expression-changed genes in two cultivated groups comparing with OG, and expression-divergent genes (CG1_CG2) into the function roles associated with the bulb traits. **d***Asa2G02184.1* and *Asa4G00255.1* show distinct difference of expression in three garlic groups. **e** Correlation between the transcript abundance of *Asa2G02184.1* and *Asa4G00255.1*, and the clove number traits in 81 accessions. The number in the top right panes represents the correlation coefficient, and the orange and blue circle indicates the negative and positive correlation, respectively; *** indicates the significance at 0.001 level. The column diagram in the diagonal panes shows the distribution of trait phenotype and expression FPKM value of genes, in which the red line indicates a distribution trend. The scatter plot in the bottom left panes shows the correlation between the transcript abundances and the clove number traits in 81 accessions, where the red line indicates a trend. The orange, green, and blue dots represent the accessions from OG, CG1, and CG2 groups, respectively

Levels of gene expression underpin organismal phenotypes, and it is often under stabilizing selection through shifts in the frequency of alleles in the domestication [20–22]. To characterize the strength of selection on gene expression in CG1 and CG2, we performed the RNA sequencing for developmental bulbs sampled from 81 accessions of OG, CG1, and CG2. After filtering the low-quality reads, a total of ~32.4–56.8 million clean reads were mapped into the garlic genome for each accession (Additional file 1: Table S12). Shannon-Wiener index (*H*’ value) was used to estimate the diversity of transcript abundance [23]. The result revealed a total of 44,598 genes expressed in the investigated population (Fig. 3a); of which, 28,538 (64.0%), 29,218 (65.5%), and 29,088 (65.2%) displayed a *H*’ value of 1.7–2.2, 2.8–3.4, and 3.0–3.7 in OG, CG1, and CG2, respectively, indicating a distinct difference in the diversity of gene expression among three garlic groups (Fig. 3a). Comparing with OG, the expression of 15.0% of garlic genes (8621 genes) showed difference in CG1, whereas this percentage in CG2 reached 17.5% (10,096 genes; Fig. 3b; Additional file 2: Fig. S6). Further correlation analysis between the transcription abundance of gene and bulb traits indicated that these expression-changed genes were significantly enriched in the function roles associated with the bulb traits, except those of CG1 for clove weight (*P* < 0.001; Fig. 3c; Additional file 1: Table S13). Notably, among these genes that underwent expression change, 97.4% and 95.6% in CG1 and CG2 were in the outside of selective genomic region, which suggested that the expression difference of most genes resulted from a regulation of selective genes, but not from direct genomic selection in their regulated region. For instance, *Asa2G00681.1*, an *early flowering 4* (*ELF4*)-like gene, showed upregulated-expression in the enlarging developmental bulbs (Additional file 2: Fig. S7); furthermore, associated transcriptomic analysis indicated a positive correlation between its transcript abundance and the clove weight in 81 transcriptome-investigated accessions (Additional file 2: Fig. S8), suggesting a role of this *ELF4*-like gene in the clove-enlarging growth. *Asa2G00681.1* displayed a distinct difference of transcript abundance between CG1 and CG2; however, selection for *Asa2G00681.1* had not been observed in both CG1 and CG2 (Additional file 2: Fig. S7).

In addition, we identified 16.3% of garlic genes (9400 genes) that showed differential expression between CG1 and CG2, including 3795 genes that are specifically expressed in either CG1 or CG2 (Fig. 3b), which suggested a great divergency in the transcriptome of two cultivated groups. Of these genes, 31.0%, 13.6%, 33.1%, and 32.1% showed an association with the clove number and weight, and bulb weight and diameters in the expression level (*P* < 0.001), respectively, which were significantly enriched in the function role for the bulb growth (Fig. 3c; Additional file 1: Table S13). This result indicated that the divergency of transcriptome was potentially linked to the difference of bulb traits in CG1 and CG2. For example, the transcript abundance of *YUUCA*-like *Asa2G02184.1* and *TOPLESS-RELATED* gene (TPR) *Asa4G00255.1* showed a significant difference between two cultivated groups (*P* =1.3×10^−13^ and 1.4×10^−15^, respectively; Fig. 3d). In morphology, cloves are the buds of garlic, and Arabidopsis *YUUCA* and *TPRs* play crucial roles in controlling the bud outgrowth [24]; correspondingly, the transcript abundance of *Asa2G02184.1* and *Asa4G00255.1* was dramatically associated with clove number in the 81 accessions (*P* = 6.3×10^−7^ and 1.4×10^−6^, respectively; Fig. 3e), indicating a potential involvement of them in the clove formation. Probably, the differential expression of *Asa2G02184.1* and *Asa4G00255.1* between CG1 and CG2 have logical roles for the diversification of clove number in these two cultivated group. Taken together, our results suggested that, after diverging and the respective evolution, the transcriptomic architecture of two cultivated garlic groups displayed distinct difference.

### Deleterious mutations underwent separate accumulation and selection-based removal in CG1 and CG2

**Fig. 4:**
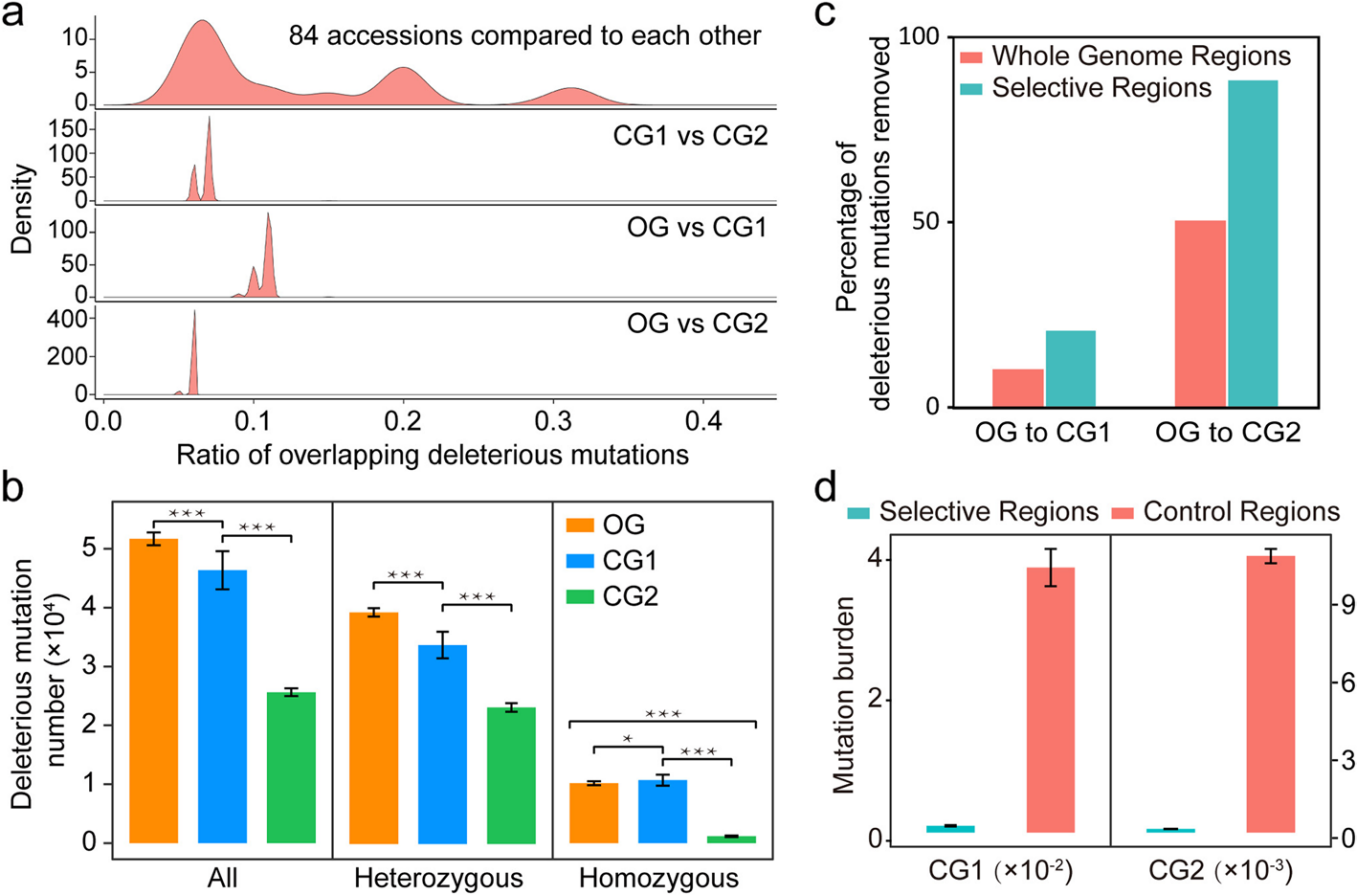
Deleterious mutations burden in garlic genome. **a** High diversity of mutation burden in 84 garlic accessions. The *x*-axis indicates the ratio of overlapping deleterious mutations in any two accessions, and the *y*-axis indicates the density of the ratio. **b** Comparison of the deleterious mutation number among OG, CG1, and CG2. Left panel represents the total of deleterious mutation number, middle panel represents heterozygous and right panel represents homozygous. Mann-Whitney *U* test was used (***: *p* < 0.001, **: 0.001 < *p* < 0.01, *: 0.01 < *p* < 0.05, data are represented as mean ± SEM). **c** Comparison of the percentage of deleterious mutations removed between the whole genome and the selective regions through comparing CG1/CG2 to OG. **d** Comparison of mutation burden between selective regions and non-selected genomic regions (Control regions). Left and right panels represent the data from CG1 and CG2, respectively

We evaluated the mutation burden in 84 garlic accessions and revealed that, among 487,215 nonsynonymous variations (Additional file 1: Table S14), 28.64% (139,518) were predicted to be deleterious. Of these deleterious mutations, there were 80.07% fewer homozygous deleterious burden than the heterozygous one, and 34.18% (47,687) were rare (allele frequency ≤ 0.05; Additional file 2: Figs. S9, S10). Investigation of the overlap of deleterious mutations between any two accessions identified a varying overlapping level between different accessions, and the distribution of overlap ratios showed three distinct peaks in 6.51%, 19.78%, and 31.42%, respectively; furthermore, only average of less than 9% of predicted deleterious substitutions were overlapped between the accessions of intergroups (Fig. 4a, Additional file 1: Table S15). These results indicated that three garlic groups underwent separate accumulation of deleterious burden.

Garlic is entirely sterile, which causes a challenge to remove their deleterious mutations by recombination. We observed a distinct reduction in CG1 (46,349) and CG2 (25,624) by comparing with OG (51,681), indicating that there still was a remission in the mutations burden in the cultivated garlic (Additional file 1: Table S16). Additionally, more homozygous deleterious mutations in CG1 and less those in CG2 than in OG were observed (Fig. 4b), further supporting a separate evolution of deleterious burden in garlic populations. To understand the potential mechanism that drove the remission of mutations burden in cultivated garlic, we analyzed the mutation burden of selective regions and identified 21.02% of deleterious substitutions that were removed in the selective regions of CG1, which was higher than the average ratio in the whole genome of CG1 (10.32%; Fig. 4c); consequently, the density of deleterious substitutions in the selective regions was 97.43% fewer than in the other genomic regions of CG1 (Mann-Whitney *U* test, *P* = 3.3 ×10^−13^; Fig. 4d). Similar results have been found in CG2, that is 88.61% of deleterious substitutions were removed in the selective sweeps, resulting in 98.66% fewer density of deleterious mutations in the selective regions than in the other genomic regions of CG2 (Mann-Whitney *U* test, *P* = 1.5 ×10^−14^; Fig. 4c, d). Therefore, our results indicated that evolutionary selection relieved the deleterious burden in clonal-propagated garlic, and the relieved degree showed distinct difference in the two investigated groups.

### Diversification of bulb traits from the differential selection in CG1 and CG2

**Fig. 5:**
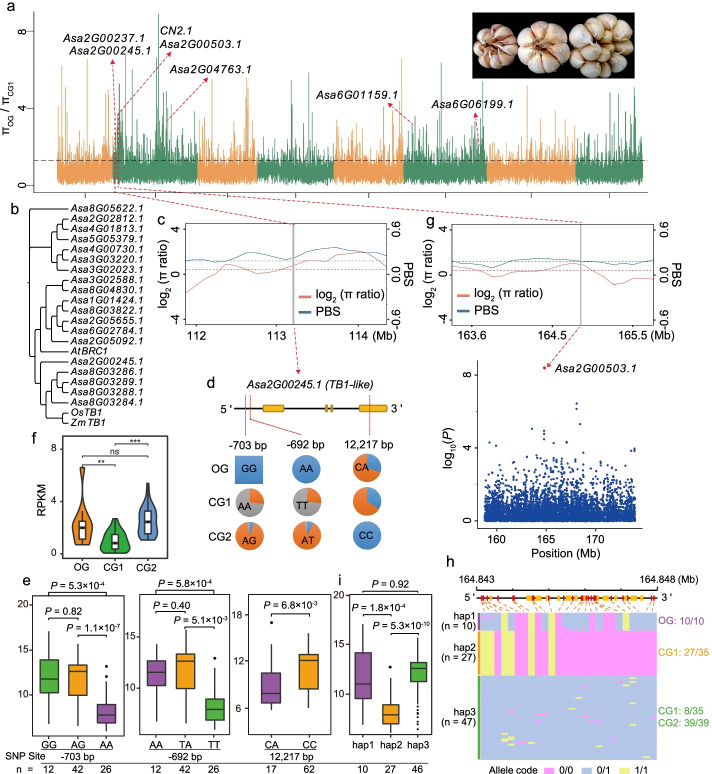
Selection signatures for the clove number in CG1. **a** Genomic regions with the top 5% of *π*_OG_/*π*_CG1_ values. Horizontal dashed lines indicate the genome-wide thresholds of the selection signals from the analysis of *π*_OG_/*π*_CG1_ values. Candidate genes/genetic loci identified in this study and overlapped with selective sweeps are marked. In the top right position of figure, bulbs from left to right represent the one from accession of OG, CG1, and CG2, respectively. **b** Phylogenetic tree of garlic TB1 homologs and AtBRC1 (Arabidopsis), OsTB1 (rice), and ZmTB1 (maize). **c** Distribution of the ratio of nucleotide diversity (*π*_OG_/*π*_CG1_) and *F*_ST_ values in the region of 112.0–114.0 Mb on chromosome 2, where *Asa2G000245.1* is located (green line). **d** Important variations of *Asa2G00245.1* and their allelic frequency in three garlic groups. Two variations in the promoter region (site of −703 and −692 bp, respectively) and one deleterious mutation (site of 12,217 bp) generated a termination codon are shown by red vertical lines. At the site of −703 bp, blue, grey, and orange part in each pie graph indicated the allelic frequency of GG, AA, and AG in corresponding garlic group, and at the site of −692 bp, they represented the allelic frequency of AA, TT, and AT, whereas at the site of 12,217 bp, blue and orange part in each pie graph indicate the allelic frequency of CC and CA in corresponding garlic group. **e** Clove number of accessions with different allele of *Asa2G000245.1*. **f** Expression level of *Asa2G000245.1* in the enlarging bulbs of three garlic groups based on the estimation of FPKM value. ns indicates no significant difference, and ** and *** indicate the significant difference at the level of 0.01 and 0.001, respectively. **g** Local Manhattan plot showing the 160–170-Mb regions on chromosomes 2. The red dot represents the SNP associated with the clove number trait, and the green arrow indicates the position of *Asa2G00253.1*. Distribution of the ratio of nucleotide diversity (*π*_OG_/*π*_CG1_) and *F*_ST_ values in the region of 163.5–165.5 Mb indicate the associated signal and *Asa2G00253.1* are in the sweep region of CG1. **h** Frequency of three haplotypes identified in the *AXR1*-like *Asa2G00253.1*. **i** Clove number of accessions with different haplotypes in *Asa2G00253.1*. The *y*-axis in the **e** and **i** figure indicates the trait value of clove number

Clove number is one of the basic components of the bulb yield of garlic, and it displayed a significant decrease in CG1 (Fig. 5a), but slight increase in CG2, comparing with OG, indicating a potential difference in the trait evolution in two cultivated groups. It is known that clove is an enlarged bud and that the outgrowth of a bud is controlled by a regulatory network where *TB1* and its ortholog (such as rice *OsTB1* and Arabidopsis *BRC1*) serve a central coordinating role [25]. There were 12 genes encoding the homologs of TB1/OsTB1 or BRC1 in garlic genome (Fig. 5b), and one of which, *Asa2G00245.1*, underwent distinct selection in CG1 (Fig. 5c). Variation investigation identified a mutation generated a termination codon in this *TB1*-like gene, and accessions carrying heterozygous allele in this mutant site produced less cloves (Fig. 5d,e). Additionally, 73.5% of CG1 accessions with mutant allele in the promoter of *Asa2G00245.1* resulted in a low expression and produced less cloves (Fig. 5d–f). Associated transcriptomic analysis revealed a significant correlation between its transcript abundance and the clove number trait in 81 transcriptome-investigated accessions (*P* = 1.5 ×10^−8^; Additional file 2: Fig. S11). These evidences commonly suggested that the selective candidate *Asa2G00245.1* played an important role in the clove number trait. Notably, significant decrease in the deleterious burden in both CG1 and CG2 relative to OG was observed (Additional file 2: Fig. S12), especially in CG1, and the homozygous mutants have been entirely removed in two cultivated groups.

To identify the potential selective genes associated with clove number, we performed a genome-wide association analysis using 230 accessions and identified 22 significant association signals (Additional file 1: Table S17). Eleven genes near the associated signals (within 200 kb) showed a significant correlation with the clove number trait in their expression level (Additional file 2: Fig. S13), indicating that they were the potential candidate of corresponding associated loci. One and two associated regions showed an overlap with the sweeps of CG1 and CG2, respectively (Fig. 5, 6a). Furthermore, we identified an *AXR1*-like *Asa2G00503.1* within the association signal on chromosome 2 (Fig. 5g), and it underwent distinct selection in CG1. *AXR1* is involved in auxin-signaling pathway of Arabidopsis and acts as repressor of bud growth [25]. Expression analysis of *Asa2G00503.1* revealed more transcripts in the accessions of CG1 than in those of other two groups (Additional file 2: Fig. S14), and its expression showed a negative association with the clove number in garlic population (Additional file 2: Fig. S11), suggesting that *Asa2G00503.1* is a candidate repressor for the clove number. Three haplotypes of *Asa2G00503.1* were identified in 84 accessions, but the hap1 and hap2 only appeared in the accessions of OG and CG1, and the accessions of CG2 only carried the hap3 (Fig. 5h); correspondingly, accessions harboring hap2 produced less cloves (Fig. 5i).

**Fig. 6:**
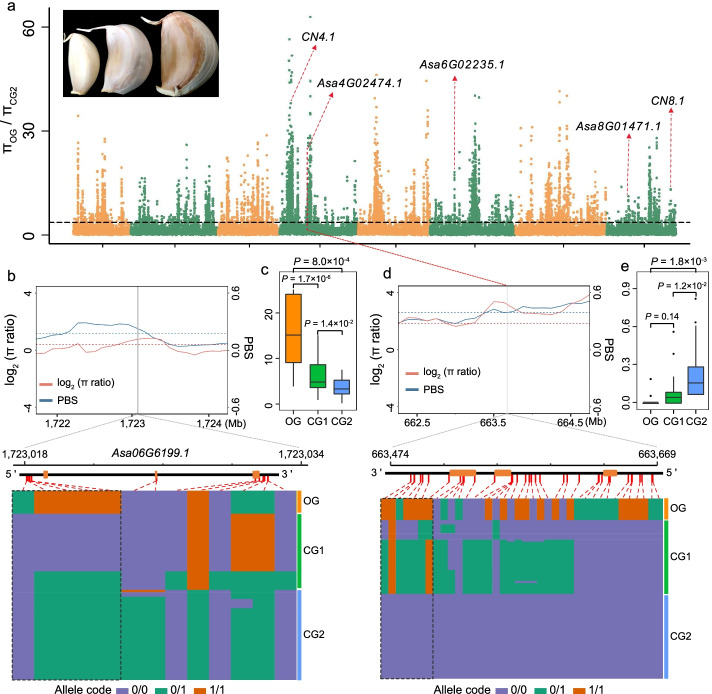
Selection signatures for the clove weight in CG2. **a** Genomic regions with the top 5% of *π*_OG_/*π*_CG2_ values. Horizontal dashed lines indicate the genome-wide thresholds of the selection signals from the analysis of *π*_OG_/*π*_CG2_ values. Candidate genes/genetic loci identified in this study and overlapped with selective sweeps are marked. In the top left position of figure, cloves from left to right represent the one from accession of OG, CG1, and CG2, respectively. **b, d** Haplotypes for the clove weight candidate gene, *Asa06G6199.1* and *Asa04G02474.1*, respectively. The black dashed box indicates the allele frequency of promoter region. **c, e** Expression levels of *Asa06G6199.1* and *Asa04G02474.1* in the enlarging bulbs of three garlic groups based on the estimation of FPKM values

Clove weight, another pivotal component of bulb yield, is affected by the clove-enlarging growth. To investigate selection signatures putatively related to the domestication of clove weight, we explored the clove-enlarging growth-related genes by comparing the transcriptome of bulbs whose cloves are before enlargement and under enlarging growth, respectively, using three garlic accessions, and identified 4658 genes with a differential expression in at least two of three examined accessions that were identified (Additional file 1: Table S18, Additional file 2: Fig. S15). In onion and potato, *Flowering locus T* (*FT*) has been identified to play central roles in controlling the enlargement growth of bulb/tuber [26, 27], and photoperiod signal stimulate the expression of *FT-*like gene to participate in the growth of potato tuber [28]. There were 38 differentially expressed genes encoding the homologs of known photoperiod-perceiving proteins, such as *CDF2*-homologous *Asa8G01471.1* and *TGA4*-like *Asa2G04763.1* and *Asa6G01159.1*, which constituted a putative model for the regulation of garlic clove-enlarging growth (Additional file 2: Fig. S16). There were 12 *FT-*like genes in garlic genome, and one of which, *Asa6G06199.1*, encoded the ortholog of onion acFT2 (Additional file 2: Fig. S17) [27] and underwent distinct selection in CG1 (Fig. 6b). Significant negative correlation between the expression level and the clove weight in garlic population suggested that *Asa6G06199.1* was a potential repressor for the clove-enlarging growth (Additional file 2: Fig. S8). Overexpression of *Asa6G06199.1* caused an early flowering in transgenic Arabidopsis (Additional file 2: Fig. S18), indicating a similar function of *acFT2* and *Asa6G06199.1*. Distinct difference in the haplotype of *Asa6G06199.1* was observed in three garlic groups. Allele of the promoter region in OG were entirely different from those in CG1 and CG2 (Fig. 6b), resulting in an expression evolution of this *FT*-like gene in two cultivated garlic groups (Fig. 6c), which should be an important reason for the clove evolution in the weight of two cultivated groups.

Gibberellins (GAs) was another important signal to modulate the expression of *FT*/*FT*-like genes [29], and seven GAs signal-related genes showed differential expression in the enlarging developmental bulbs (Additional file 2: Fig. S16), including *Asa6G01110.1*, an ortholog of potato tuber formation-related *stBEL5* (Additional file 2: Fig. S19) [30]. *Asa2G00237.1*, a homologous gene of *SLR1* encoding a DELLA protein that is known as the key factor for the GA-signal transduction of rice (Additional file 2: Fig. S20) [31], underwent selection in CG1, and its expression was observed to have a significantly negative correlation with clove weight in garlic population (*P* = 1.6 ×10^−4^; Additional file 2: Fig. S8). GA20ox is a catabolic enzyme of bioactive GAs, and the induction of *StGA2ox1* gene plays a pivotal role in the tuberization of potato [32]. Similarly, this study identified two garlic GA20ox genes, *Asa4G02474.1* and *Asa0G05638.1*, whose transcript abundance showed significant association with the clove weight in garlic population (Additional file 2: Fig. S8), suggesting their roles in the control of bulb-enlarging growth. Interestingly, *Asa4G02474.1* underwent expression evolution, thereby to express specifically in CG2 (Fig. 6e). Only one haplotype existed in CG2, and its sequence was distinctly different from those of OG and CG1, especially in the promoter region (Fig. 6d). These results indicated that the selection of *Asa4G02474.1* resulted in the emergence of its expression in CG2, which should be associated with the differentiation of clove weight between the accessions of CG2 and the other two groups.

Notably, 125 and 248 genes that underwent putative selection in CG1 and CG2, respectively, showed differential expression between the bulbs under different developmental stages, suggesting a potential involvement of them in bulb development. Of these selective genes with differential expression, only eight were subjected to common selection in both CG1 and CG2 (Additional file 1: Table S19), accounting for 6.4% and 3.2% of selective candidates of CG1 (125 genes) and CG2 (248 genes), respectively. Furthermore, among candidates for clove number and weight that were identified from genome-wide trait associations and/or associated transcriptomic analysis, although they had distinct selection signatures in evolutionary history, none of them underwent common selection in CG1 and CG2 (Additional file 2: Fig. S21). These results indicated a different selective pattern for bulb traits in these two cultivated groups, which further supported the finding of independent domestication between CG1 and CG2.

## Discussion

Genetic and breeding studies of garlic were challenged by a large and complex genome and its sterility, and the genetic architecture of traits was almost entirely unknown in this *Allium* species. In this study, we identified ~129.4 million variations in garlic genome, representing valuable resources for the genetic and breeding studies of garlic. Using these variations as a basic tool, the achievement for dissecting the genetic architecture of clove number trait represented a great progress in the genetic study of this *Allium* crop. Additionally, sterility makes a challenge to estimate the genetic distance between garlic groups by evaluating their cross-compatibility. Five taxonomic groups of garlic were proposed, including the longicuspis group, subtropical group, mediterranean sativum group, ophioscorodon group, and pekinense group (a subgroup of longicuspis group probably), of which the longicuspis group from Central Asia is considered as the basal group, and the subtropical group and pekinense mainly grow in China and other East Asian countries [7]. However, this study indicated four main garlic groups based on the genomic variations, including two groups that existed in China, CG1 and CG2. Similarly, a previous study proposed two main groups grown in China, that is, the subtropical group that was speculated to originate independently a long time ago from the longicuspis group, and the pekinense group that was consider to diverge from the longicuspis group ~1500 years ago since the introduction into North China [7]. In this study, our genomic evidences distinctly revealed that both CG1 and CG2 diverged from the ancestral group prior to the history of crop domestication by human, and because the sterility causes a reproductive isolation, they are independently domesticated.

This study found that OG accessions had a closer genetic relationship with the accessions of outgroup species, indicating OG accessions have more genetic information of wild progenitor of garlic in their genome than in the genome of accessions from the other three groups. Therefore, although the absence of wild species used in this study, OG accessions should be qualified for evolutionary study as a substitute of wild germplasms. Slightly low nucleotide diversity in OG than in other garlic groups resulted probably from a relatively small population size or a bad randomness of sample in this group. This study revealed little selective genome shared in CG1 and CG2, although three approaches were used to detect the selective signals of genome. Especially, the XP-CLR approach only identified only 1.6 Mb genome that underwent common selection in CG1 and CG2. It is known that XP-CLR is a power tool in detecting ancient selective events [33]. Because garlic is sterile and only can reproduce asexually, the ancient selective events should have been fixed in CG1 and CG2. Moreover, ancient selective events generally result from natural selection. Unlike domesticated selection that frequently focuses on some important regions associated with agronomic traits by human and that results in many common selective regions among different subpopulation of one species, natural selection causes less common selection than domesticated selection in these subpopulations. Therefore, extremely few ancient selection was commonly observed between CG1 and CG2 which further validated their independent domestication.

It is known that there are many species, including garlic, that displayed drastic decrease of population scale, even become extinct during the Younger Dryas period, because of a low temperature environment over a thousand years. Garlic cloves stored plentiful nutrition to supply the seedling; correspondingly, seedlings germinated from large clove are stronger and have a better ability to resist the cold environment. Although accessions of both CG1 and CG2 showed a large clove of bulb, only the accessions of CG1 can overcome the low temperature environment during the Younger Dryas period, to make its population scale expand in that time, whereas the population size of CG2 decline persistently until ~7000 YBP when human have begun to perform the crop domestication. Probably, the large size of clove in the CG1 accessions derived from a natural selection for the purpose of cold environment adaption, whereas the well performance of clove in the CG2 accessions results from a domesticated selection by human. This hypothesis can well explain the differential evolutionary pattern for the clove weight trait in CG1 and CG2. In summary, this study provided us important insights into the evolutionary history of garlic.

## Conclusions

We identified 120,857,927 SNPs and 8,551,841 indels in garlic genome by resequencing of 84 germplasms, representing a valuable resource for the studies of biology and breeding in this economically important crop. Based on the genome-wide variations, we explored the evolutionary history of garlic and found that two groups cultivated in China domesticated independently. Furthermore, we revealed that independent domestication leads to distinct difference of transcriptomic architecture, few overlaps of deleterious substitutions, and differential selections for the bulb traits in these two garlic groups. These findings provide important insights into the evolutionary history of this Allium crop.

## Methods

### Plant materials

Garlic accessions of four researching teams from Institute of Bast Fiber Crops (Chinese Academy of Agricultural Sciences, China), Shandong Agricultural University (China), Northwest A&F University (China), and Shandong Research Center of garlic Engineering (China) were collected, and the redundant accessions were removed. Finally, a diverse collection of 230 garlic accessions and 3 accessions of *Allium ampeloprosum* were obtained. Information about the accessions, including the collected site, country of origin, group, identity of the variety, and GBS sequencing data summary, is provided in Additional file 1: Table S1. All these accessions were grown in two locations of Shandong Province, Jinxiang and Tai’an in 2018 and 2019. For each accession, thirty individuals were used to count the clove number.

### DNA isolation and genome sequencing

Genomic DNA (gDNA) was extracted from fresh leaves of a single plant of each accession using the DNAsecure Plant Kit (TIANGEN). The gDNA quality was checked using agarose gel electrophoresis and concentration was quantified using Qubit 2.0 Flurometer (Life Technologies, CA, USA). At least 1.5 μg of gDNA was used to construct a sequencing library with the TruSeq Nano Sample Prep Kit (Illumina Inc., San Diego, CA, USA) according to the manufacturer’s specifications. In details, the libraries were prepared following these steps: the genomic DNA sample was fragmented by sonication to a size of ~350 bp, then DNA fragments were end-polished, A-tailed, and ligated with the full-length adapters for Illumina sequencing with further PCR amplification. As for the GBS libraries, the genomic DNA was digested using the restriction enzyme *MseI* (New England BioLabs, Ipswich, MA, USA), and then the fragments were ligated to the adapters with barcodes and performed for PCR amplification. At last, PCR products were purified (AMPure XP bead system) and libraries were analyzed for size distribution by Agilent2100 Bioanalyzer and quantified using real-time PCR. Subsequently, sequencing data with 150-bp read length were generated using the Illumina Hiseq X platform.

### Mapping and variant calling

To avoid reads with artificial bias, i.e., low-quality paired reads, which primarily result from base-calling duplicates and adaptor contamination, we removed the low-quality reads using the package FastQC (v.0.11.9) (https://www.bioinformatics.babraham.ac.uk/projects/fastqc/) with the default parameters. The high-quality reads were aligned to the garlic reference genome (GCA_014155895.2) using Burrows-Wheeler Aligner (BWA) software (v.0.7.8) [34], with the parameters of “mem -t 8 -k 32 -M.” The Alignment results were then converted into BAM format and sorted using the SAMtools (v.1.3) [35]. PCR duplicate reads were removed with SAMtools. If multiple read pairs had identical external coordinates, only the pair with the highest mapping quality was retained. Subsequently, the genomic variants for each sample were identified using the Bayesian approach implemented in the package SAMtools. The variants were further filtered using the following criteria: depth for each individual < 3, genotype quality for each individual < 5, QUAL < 20. For each individual of 84 whole-genome resequencing, we extracted SNPs for subsequent deleterious mutation prediction. We screened out population variants after filtering minor allele frequency (MAF) < 0.05 and population missing ratio > 0.2. All the identified SNPs were then annotated using the package ANNOVAR [36], according to the gene models of garlic genome, and were categorized based on their positions, including exonic regions, intronic regions, intergenic regions, splicing sites, and 1-kb upstream and downstream regions. The variants in coding regions were further categorized based on their functional effects, including synonymous, nonsynonymous, frameshift, non-frameshift, stop gain, and stop loss.

### Analysis of population structure and population diversity

To clarify the phylogenetic relationships among all the accessions, a NJ tree was constructed based on the *p*-distance using the program TreeBest (v1.92) [37], with 1000 bootstrap iterations. PCA was performed using the package GCTA [38]. The significance level of eigenvectors was determined using the Tracy-Widom test. Population structures were investigated using the ADMIXTURE program (version: 1.3.0) [39], and each *K* value was run 10 times with varying random seeds; the Q-matrices were aligned using software pong (version: 1.4.7) [40] and clustered based on the similarity. Then, the matrices belonging to the largest cluster were averaged to produce the final matrix of admixture proportions. To calculate the population diversity, we first screened and removed windows with less than five SNPs. Subsequently, the package VCFtools (v0.1.14) [41] was used to estimate the nucleotide diversity (*θπ*) [42] within each group and the *F*_ST_ between the two groups [43]. Nucleotide diversity was calculated based on 500-kb sliding windows, using genome-wide SNPs. For GBS SNPs, because of a low saturation of them in genome, a windowless model was applied to assess genome-wide nucleotide diversity using them, which effectively avoids the inaccuracy of nucleotide diversity that is caused by numerous windows with few SNPs.

### Demographic history estimation

The per-generation nucleotide mutation rate (*μ*) is firstly estimated by calculating the synonymous divergence (*Ks*) values for all pairs of the single-copy orthologous genes between garlic and *Asparagus officinalis* using the basic model in PAML package [44], according to the formula: *μ* = *Ks*/2*T*, where *T* is the estimated divergence time (80.8 Mya) [11]. Subsequently, we inferred the fluctuation of the effective population size for garlic accessions with SMC++ (v1.15.2) [45] based on a constant generation time of 1 year and *μ* value of 3.84×10^−9^.

### Genome-wide selective sweep scanning

We employed several statistical methods to identify genome-wide selection signals based on the SNP set. Firstly, by comparing the pairwise *F*_ST_ between designed compared patterns with a sliding window (500-kb windows sliding in 125-kb steps), we employed Population Branch Statistic (PBS) approach to detect incompletely selective sweeps over short divergence times [46]. Our approach designed to take advantage of outgroup and used to identify selection targeted on the tested lineage. The PBS was calculated as follows:

$$\left({T}^{TP- Cp}+{T}^{TP- CO}-{T}^{TP- CO}\right)/2$$

where *T* represents the population divergence time in units scaled by the population size, which is the negative log transformed (1 − *F*_ST_) between two populations. TP represents the targeted population, CP indicates the control population, and CO implies the outgroup. We considered the window as the candidate selected regions when PBS value of the comparative sliding windows at a significance of *P* < 0.05 (*Z-*test). Specifically, for comparative pattern of CG1 via OG, the design formula is (*T*^CG1 − OG^ + *T*^CG1 − CG2^ − *T*^OG − CG2^)/2 ; for the pattern of CG2 via OG, the formula is (*T*^CG2 − OG^ + *T*^CG2 − CG1^ − T^OG − CG1^)/2.

Secondly, the test of cross-population composite likelihood ratio (XP-CLR; https://github.com/hardingnj/xpclr) [33] was performed with the following parameters: sliding window size of 0.01 cM, grid size of 50 k, maximum number of SNPs within a window is 100, and correlation value for two SNPs weighted with a cutoff of 0.95. The windows with top 5% XP-CLR score were taken as outliers.

Thirdly, nucleotide diversity (*θπ*) were calculated based on a sliding window (500-kb windows sliding in 125-kb steps) in two populations, A and B. The statistic log_2_ (*θπ*_A_ / *θπ*_B_) was then calculated with respect to A and B populations. An unusually negative value (5% outliers) suggests selection in population A, and the top positive value (5% outliers) indicates selection in population B.

The genetic diversity *π* is a classic statistic to detect signals of selection (especially hard sweeps) by assuming that selected regions show a reduced genetic diversity. PBS method can be viewed as a model-based extension of *F*_ST_, which is very powerful in detecting incomplete selective sweeps over short divergence times [45], while the XP-CLR method is able to detect ancient selective events [33]. Thus, the three methods are compatible and complementary. Finally, a total of 143.0 Mb, 78.0 Mb, and 1.6 Mb genome with selective signals were detected by *θπ* ratio, PBS, and XP-CLR, respectively. As the three methods are good at detecting different selective events, we took the candidates with support from at least two statistics. Subsequently, all candidate regions were assigned to corresponding SNPs and genes.

### Deleterious mutations prediction

We predicted the amino acid substitutions and their effects on protein function for nonsynonymous SNPs of 84 garlic core accessions applying the SIFT algorithm [47]. The amino acid substitution is predicted to be deleterious if the score is ≤ 0.05 and tolerated if the score is > 0.05. Of the deleterious mutations, if the genotype in an individual is homozygous and is different from the referenced genome, we define it to be a homozygous deleterious mutation; if the genotype in an individual is heterozygous, and one of its allele is different that of referenced genome, we define it to be a heterozygous deleterious mutation. According to a previously reported method [48], the ratio of overlapping deleterious mutations between any two accessions was calculated using the following formula:

$$\mathrm{Ratio}=\frac{a+0.5b+0.25c}{d}$$

where *a*, *b*, and *c* indicate the number of deleterious mutations that are homozygous in reference genotype, heterozygous, and homozygous in allele genotype, respectively; *d* indicates the total number of deleterious mutations in two accessions.

### RNA sequencing

The 81 garlic accessions were grown in the farm of Shandong Research Center of Garlic Engineering, Jinxiang, China, in Oct. of 2019, and in the next April, their developmental bulbs were sampled. For each accession, bulbs of five plants were pooled as a sample. In addition, three varieties, Chalingzipisuan, Ershuizao, and Yuanjiangyangsuan, were planted in the farm of Institute of Bast Fiber Crops, Chinese Academy of Agricultural Sciences, China, in Oct. of 2019, and in Feb. and Mar. of 2020, when the bulbs had not initiated to enlarge and were under enlarging growth, respectively, the parts of bulbs were collected. Three individuals were used as three replications. These samples were immediately frozen in liquid nitrogen and stored at −80 °C until use.

Total RNAs were extracted using the Plant RNA extraction Kit, and then were used for constructing the cDNA libraries with a fragment length of 300 bp using a NEBNext® UltraTM RNA Library Prep Kit for Illumina® (New England BioLabs, Ipswich, MA, USA) following the manufacturer’s instructions. Paired-end sequencing was performed for each library using a Hiseq X platform. After trimming the adapter sequences and filtering the low-quality reads, the clean reads were generated for further use.

### Expression analysis

To quantify the transcript abundance in the RNA-sequencing libraries, clean reads from each library were aligned with the garlic reference (GenBank accession: GCA_014155895.2) using hisat2 software (version: 2.2.1.0) [49], with default parameters. The fragments per kilobase per million reads (FPKM) [50] were estimated to quantify the expression level of each gene. Differentially expressed genes between the bulbs under two different growth stages in Chalingzipisuan, Ershuizao, and Yuanjiangyangsuan were determined using the DEseq program (version: 1.18.0) [51], and the expression with more than twofold difference were deemed to be significant (*P* < 0.05). Genes showed expressed differences in two of three investigated varieties were considered as differentially expressed genes with high confidence.

The 81 accessions subjected to RNA sequencing were derived from three groups, i.e., OG, CG1, and CG2, and the detailed RNA sequencing data summary has been provided in Additional file 1: Table S12. For each accession, at least 30 million reads that were mapped into garlic reference were requested. Expression difference of each gene between groups was estimated using the analysis of variance (ANOVA), and the false discovery rate (FDR) was estimated by multiple testing of the *P* value using the Benjamini–Hochberg procedure, and genes with an FDR less than 0.05 were deemed to be significantly differentially expressed genes between groups. If one gene has not any transcript observed in the libraries of accessions of OG, but expresses in more than 90% of accessions of CG1 or CG2, we defined that it emerges expression during garlic evolutionary history. Conversely, if one gene expresses in more than 90% of OG accessions, but there was not any transcript observed in the libraries of accessions of CG1 or CG2, we defined that it loses expression during evolutionary history.

The diversity of transcript abundance of each gene in 81 accessions was estimated by the Shannon-Wiener index (*H*’ value) [23, 52]. To estimate the *H*’ value of transcript abundance for one gene, its average abundance of transcript (*x*) and standard error (*δ*) were calculated in each accession. Then, ten intervals of value were set, with a value difference of 0.5δ, namely, the first interval was “*X*_*1*_ < (*x*−2*δ*),” and the second one was “(*x*−2*δ*) ≤*X*_*2*_ < (*x*−1.5*δ*),” and so on, and the last interval was *X*_*10*_ > (*x*+2*δ*).” Subsequently, the frequency of accession in each interval was counted, using the number of accessions with transcript abundance that fell into the corresponding interval divided by total number of accessions. Finally, the *H’* value of transcript abundance for each gene was calculated using the following formula:

$${H}^{\prime }=-\sum pi{\log}_2 pi$$

where *pi* is the frequency of accession in the *i*th interval.

Association of transcript abundance with phenotypic value of four bulb traits was determined by performing the Pearson correlation analysis in 81 transcriptome-investigated accessions. To determine whether expressed-changed genes between the groups were enriched in the function associated with four bulb traits, genes that showed significant correlation with traits from expression-changed genes and all expressed garlic genes were counted, which have been listed in Additional file 1: Table S13. Then, based on the number of correlated genes and total gene in the set of expression-changed genes and all genes, Fisher’s exact test was used to calculated the *P* value, and an *P* value with less than 0.001 to be considered as a significant enrichment.

### GWAS

All 9,403,677 SNPs (MAF ≥ 0.01, missing rate ≤ 0.5) identified from the GBS sequencing of 230 garlic accessions were used to perform GWAS of clove number trait, based on the efficient mixed model association expedited (EMMAX) program [53]. Population stratification and hidden relatedness were determined using a kinship (*K*) matrix generated using the FaST-LMM program [54]. The *P* value threshold for suggestive association loci was set to 5.32 × 10^–9^, according to the estimation from Bonferroni correction based on the effective number of independent markers [55]. Because of low density of gene in garlic genome (average 282 kb intergenic distance), genes near to the associated signals (within 200 kb) were further analyzed for their expression.

### Overexpression of Asa6G06199.1

Full-length sequence of gene was amplified from a garlic cDNA library by high-fidelity PCR (primer sequences listed in Additional file 1: Table S20 and was ligated into the PBI121 vector, thereby to generate a construct in which CaMV 35S promoter drive an overexpression of *Asa6G06199.1*. Subsequently, the constructed plasmid was introduced into *Agrobacterium tumefaciens* strain GV3101 based on the heat-shock method, and the resultant *Agrobacterium* was infiltrated into Arabidopsis using the floral dip method [56]. Transgenic plants were grown in a greenhouse under conditions of 22 °C and a 9/15-h light/dark photoperiod regime.

### Statistical analysis and phylogenetic analysis of candidate protein

Statistical analysis was performed with the SPSS software. Phylogenetic analysis of candidates and their homologs of other plants was performed with two steps: sequence alignment was carried out using the Clustal program [57], and that an unrooted phylogenetic tree was constructed using the MEGA software using the neighbor-joining (NJ) method and the bootstrap test carried out with 1000 replicates [58].

## Supplementary Information

Additional file 1: Table S1. Basic information for 233 accessions. Table S2. Statistics of variation in 230 garlic accessions from the GBS sequencing. Table S3. Basic information for 84 resequenced accessions. Table S4. Statistics and distribution of SNPs identified from 84 garlic accessions by resequencing. Table S5. Statistics and distribution of small indels identified from 84 garlic accessions by resequencing. Table S6. Divergent genomic regions between CG1 and CG2 (*F*_*ST*_ > 0.5). Table S7. Genomic region with top 5% θ*π* ratio. Table S8. Genomic region with top 5% population branch statistic (PBS) values. Table S9. Genomic region with top 5% XP-CLR values. Table S10. Genes located in the sweep region of CG1 and CG2. Table S11. Genes underwent selection in both CG1 and CG2 groups. Table S12. Statistical data for RNA sequencing of bulbs in 81 accessions. Table S13. Statistical analysis for genes associated with bulb traits. Table S14. Statistical data of SNPs in the genic regions of 84 accessions. Table S15. Statistics of overlap ratio for the deleterious mutations between accessions. Table S16. Summary of deleterious mutation burden in 84 accessions. Table S17. Genetic loci associated with clove number in 230 accessions (*P* < 5.32 ×10^−9^). Table S18. Differentially expressed genes by comparing the profiling of bulbs whose cloves are before enlargement and under enlarging-growth. Table S19. Differentially expressed genes underwent common selection in CG1 and CG2. Table S20. Primer sequences for constructing the overexpressed vector of *Asa6G06199.1.*

Additional file 2: Figure S1. The average proportion of reads with different alignment lengths by mapping the garlic reference in 84 accessions. Sequence reads generated from whole genome resequencing. Figure S2 Distribution of garlic variations. Track i indicates the density of SNPs from Genotype-by-sequencing (GBS), and track ii and iii indicate the density of SNPs and indels from whole-genome sequencing (WGS). Figure S3. The bulbs from three varieties, S41, S59, and S56, which were the accessions of OG, CG1, and CG2, respectively. Figure S4. Effective population size *Ne* of four garlic populations estimated using the GBS SNPs by the SMC++ method. Figure S5. A Venn figure shows the selective gene number by the population branch statistic (PBS, green), the θπ ratio (π_OG_/π_CG_, blue), and the test of cross-population composite likelihood ratio (XP-CLR, yellow). Figure S6. Comparison of gene expression level among three groups. Manhattan plot shows the *P* value for the difference of transcript abundance of each gene between two groups by ANOVA analysis. The y axis indicates the -log10(*P* value). Figure S7. Characterization of *ELF4*-like *Asa2G00681.1*. a, Expression level of *Asa2G00681.1* in the bulbs under enlarging growth (blue column) and un-enlargement (orange column) in three varieties. b, Expression level of *Asa2G00681.1* in the enlarging bulbs of three garlic groups based on the estimation of FPKM value. In the Figure a and b, the y axis indicates the FPKM value, and Ns indicates no significant difference, and ** and *** indicate a significant difference at the level of 0.01 and 0.001, respectively. c, Distribution of the ratio of nucleotide diversity (π_CG_/π_OG_) and *F*_ST_ values in the region of 202.0-204 Mb on chromosome 2. The grey line indicates the position of *Asa2G00681.1*. Figure S8. Correlation between the transcript abundance of five selective candidates and the clove weight traits in 81 accessions. The number in the top right panes (green dashed box) represents the correlation coefficient, and the red and blue circle indicates the negative and positive correlation, respectively; *** indicated a significance at 0.001 level. The column diagram in the diagonal panes shows the distribution of trait phenotype and expression FPKM value of genes, in which the red line indicates a distribution trend. The scatter plot in the bottom left panes (purple dashed box) shows the correlation between the transcript abundances and the clove weight traits in 81 accessions, where the red line indicates a trend. Figure S9. Number of heterozygous and homozygous deleterious mutations in 84 garlic accessions and three groups (OG, CG1, CG2). Each box represents the mean and interquartile range. Figure S10. Distribution of deleterious allele frequency in 84 garlic accessions. Figure S11. Correlation between the transcript abundance of three selective candidates and the clove number traits in 81 accessions. The number in the top right panes (green dashed box) represents the correlation coefficient, and the red and blue circles indicate negative and positive correlation, respectively; ** and *** indicate a significance at 0.01 and 0.001 level. The column diagram in the diagonal panes shows the distribution of trait phenotype and expression FPKM value of genes, in which the red line indicates a distribution trend. The scatter plot in the bottom left panes (purple dashed box) shows the correlation between the transcript abundances and the clove number traits in 81 accessions, where the red line indicates a trend. Figure S12. Comparison of deleterious mutations burden in the coding region of *Asa2G00245.1* among OG, CG1 and CG2. Figure S13 Whole genome association analysis of the clove number trait in 230 garlic accessions. Purple arrows indicate the associated position with significant signal (*P* < 5.32 ×10^−9^). The gene under associated loci indicates that it is near to the associated signal (within 200 kb), and its expression shows the significant correlation with the clove number trait in 81 transcriptome-examined accessions, and is considered as the candidate of corresponding loci. *, **, and *** represent the significant association at the level of 0.05, 0.01, and 0.001, respectively. Figure S14. Expression level of Asa2G000503.1 in the enlarging bulbs of three garlic groups based on the estimation of FPKM value. *, and ** indicate the significant difference at the level of 0.05 and 0.01, respectively. Figure S15. Differentially expressed genes (DEGs) in the bulbs whose cloves are before enlargement and under enlarging-growth in three varieties, Ershuizao (a), Chalingzipisuan (b), and Yuanjiangyangsuan (c). Orange dots represent genes with more transcripts in the library of enlarging bulbs, green dots represent genes with fewer transcripts in the library of enlarging bulbs, and grey dots indicate genes whose expression are not changed significantly. d, Venn diagram of differentially expressed genes from three varieties. Figure S16. A putative model for clove enlarging growth is proposed according to the orthologous analysis, in which 38 and 7 differentially expressed genes are the homologous genes involved in the photoperiod and gibberellin signal pathway. Heatmap shows the expressed difference of candidates between the bulbs whose cloves are under enlarging-growth (EB) and un-enlargement (UB). Figure S17. Phylogenetic tree of FT and FT-like proteins from six species, including garlic (blue), onion (green), Arabidopsis (purple), rice (orange), potato (black), and tomato (pink). Figure S18. Comparison of the flowering time between wild (right) and *Asa06G06199.1*-overexpressed Arabidopsis (left). Figure S19. Phylogenetic relation of garlic BEL1-like Homeodomain (BELL) members and Arabidopsis and tomato homologs, AtATH1, AtPNY, AtPNF, and StBEL5. The proteins of garlic, Arabidopsis and tomato are indicated by blue, green, and pink circle. Figure S20. Phylogenetic tree of DELLA proteins from three species, including garlic (blue), Arabidopsis (green), and rice (orange). Figure S21. Distribution of the ratio of nucleotide diversity (*π*_CG_/*π*_OG_) and *F*_ST_ values in the region near to the bulb traits-related candidates, which underwent selection in either CG1 or CG2.

Additional file 3:. Review history.

## Data Availability

The reported variation data from GBS and whole-genome sequencing have been deposited in the Genome Variation Map (GVM) in Big Data Center, Beijing Institute of Genomics (BIG), Chinese Academy of Science, under the project accession PRJCA006629 [59]. The sequence reads of transcriptome and the expression data have been deposited in the NCBI GEO database under the accession numbers GSE186042 [60] and GSE211495 [61]. The garlic assembly that was used as the reference sequence for SNP calling and expression quantifying was downloaded from NCBI database under the accession number PRJNA60638 [13].
