# Supplementary material for: Genomic insights into the evolutionary history and diversification of bulb traits in garlic
Source: Genome Biol. 2022 Sep 7;23:188. doi: 10.1186/s13059-022-02756-1 (PMC9450234; doi:10.1186/s13059-022-02756-1)
Supplement: Supplementary file 2 — Additional file 2: Figure S1. The average proportion of reads with different alignment lengths by mapping the garlic reference in 84 accessions. Sequence reads generated from whole genome resequencing. Figure S2 Distribution of garlic variations. Track i indicates the density of SNPs from Genotype-by-sequencing (GBS), and track ii and iii indicate the density of SNPs and indels from whole-genome sequencing (WGS). Figure S3. The bulbs from three varieties, S41, S59, and S56, which were the accessions of OG, CG1, and CG2, respectively. Figure S4. Effective population size Ne of four garlic populations estimated using the GBS SNPs by the SMC++ method. Figure S5. A Venn figure shows the selective gene number by the population branch statistic (PBS, green), the θπ ratio (πOG/πCG, blue), and the test of cross-population composite likelihood ratio (XP-CLR, yellow). Figure S6. Comparison of gene expression level among three groups. Manhattan plot shows the P value for the difference of transcript abundance of each gene between two groups by ANOVA analysis. The y axis indicates the -log10(P value). Figure S7. Characterization of ELF4-like Asa2G00681.1. a, Expression level of Asa2G00681.1 in the bulbs under enlarging growth (blue column) and un-enlargement (orange column) in three varieties. b, Expression level of Asa2G00681.1 in the enlarging bulbs of three garlic groups based on the estimation of FPKM value. In the Figure a and b, the y axis indicates the FPKM value, and Ns indicates no significant difference, and ** and *** indicate a significant difference at the level of 0.01 and 0.001, respectively. c, Distribution of the ratio of nucleotide diversity (πCG/πOG) and FST values in the region of 202.0-204 Mb on chromosome 2. The grey line indicates the position of Asa2G00681.1. Figure S8. Correlation between the transcript abundance of five selective candidates and the clove weight traits in 81 accessions. The number in the top right panes (green dashed box) represents the [file 13059_2022_2756_MOESM2_ESM.docx]

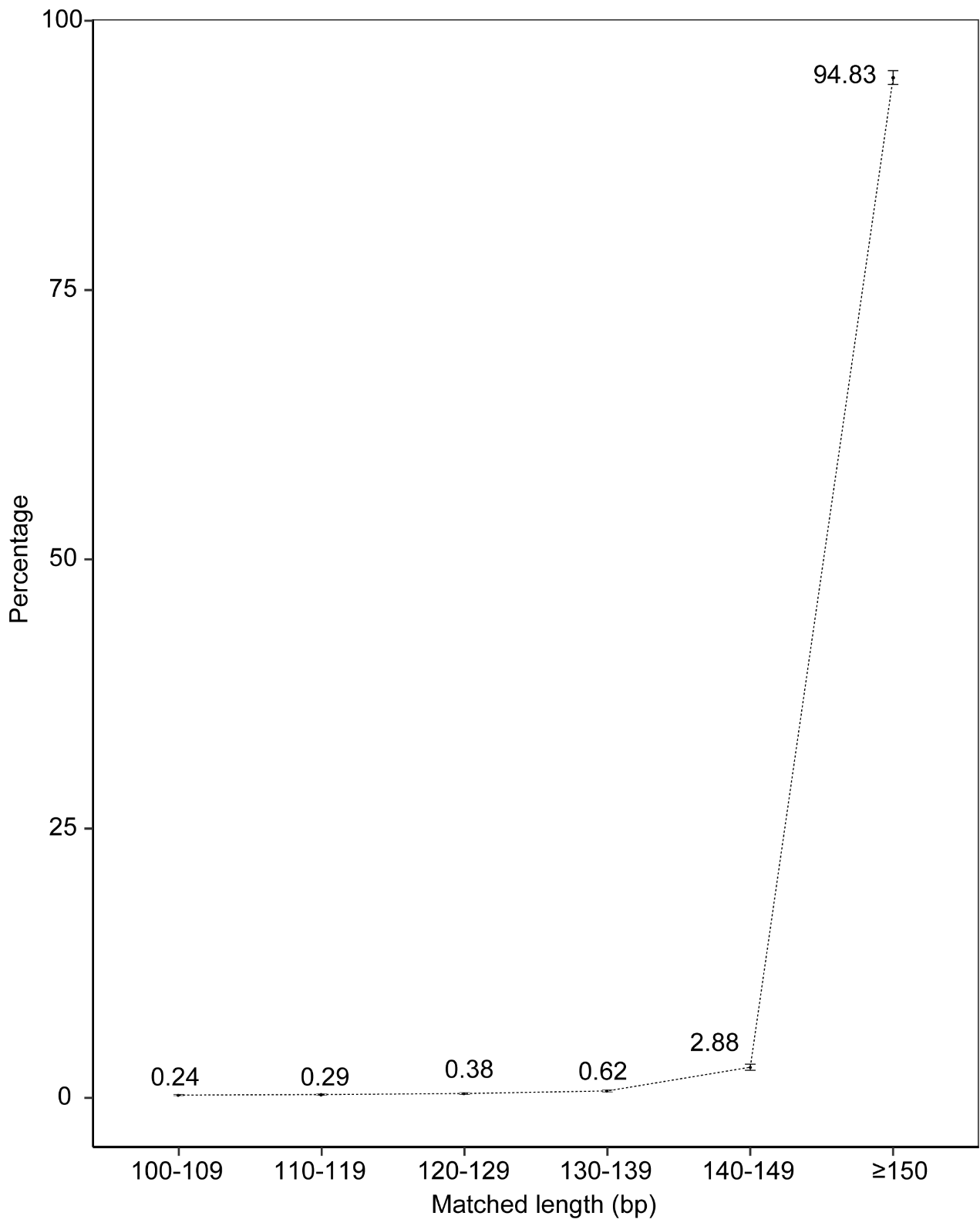


**Fig. S1** The average proportion of reads with different alignment lengths by mapping the garlic reference in 84 accessions. Sequence reads generated from whole genome resequencing.

**
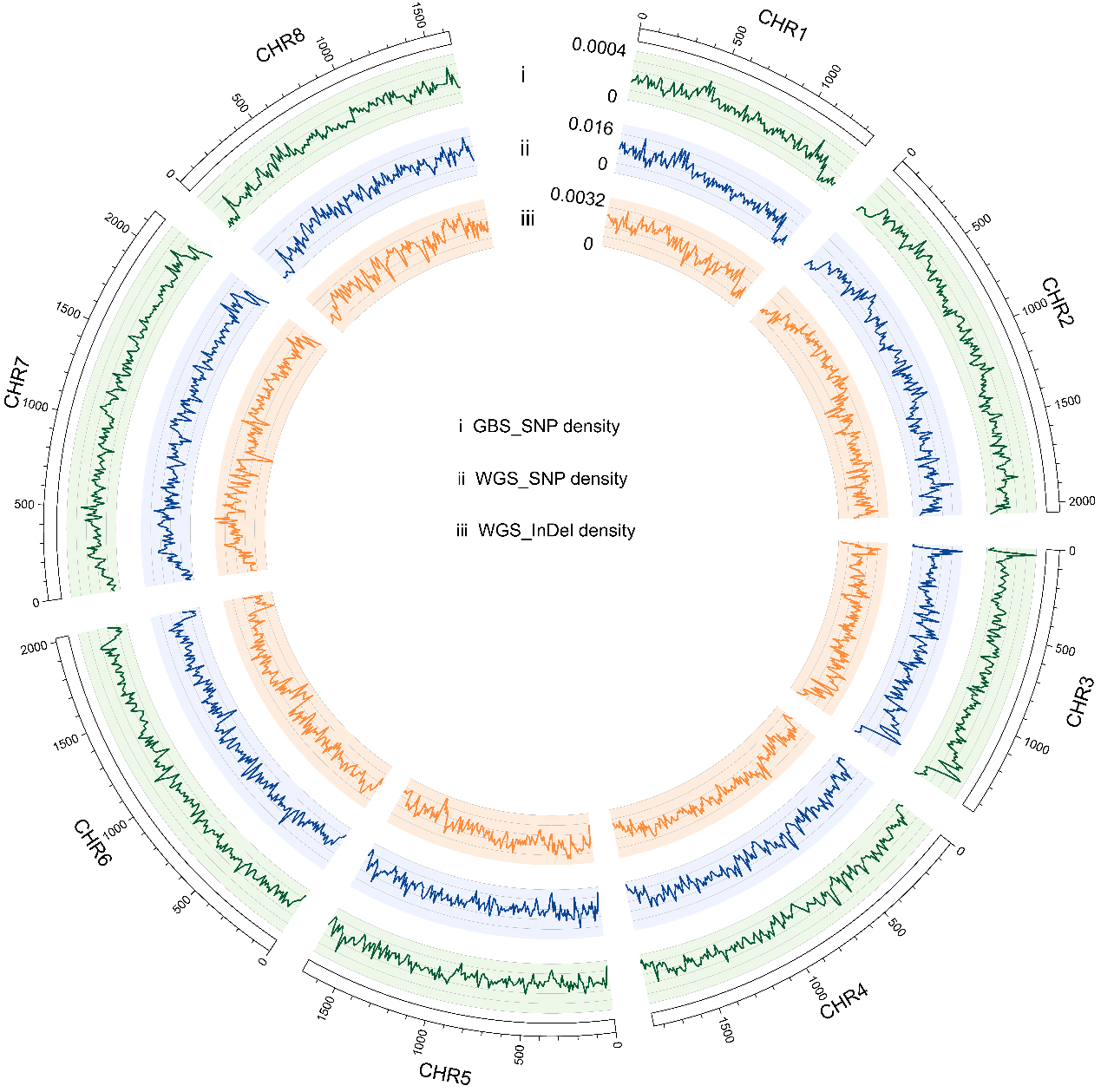
**

**Fig. S2** Distribution of garlic variations. Track i indicates the density of SNPs from Genotype-by-sequencing (GBS), and track ii and iii indicate the density of SNPs and indels from whole-genome sequencing (WGS).


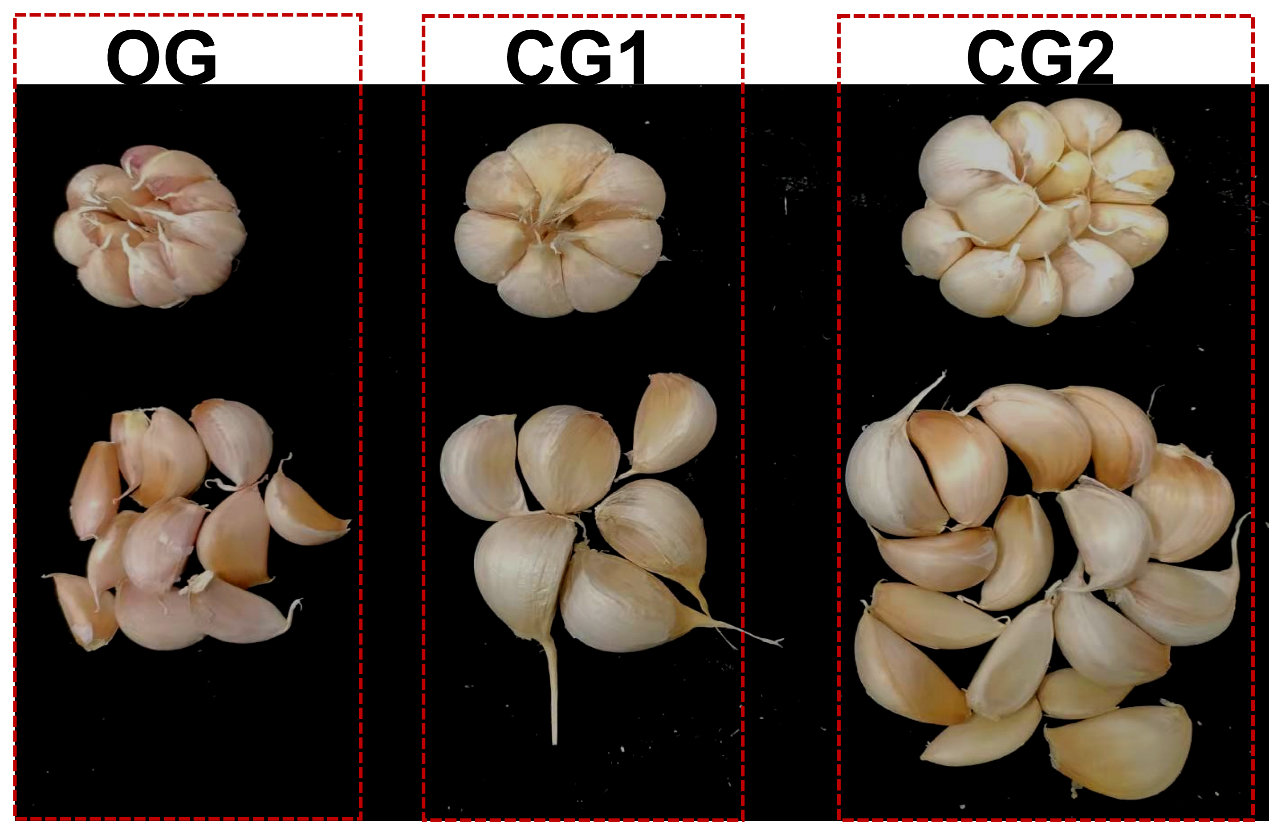


**Fig. S3** The bulbs from three varieties, S41, S59, and S56, which were the accessions of OG, CG1, and CG2, respectively.


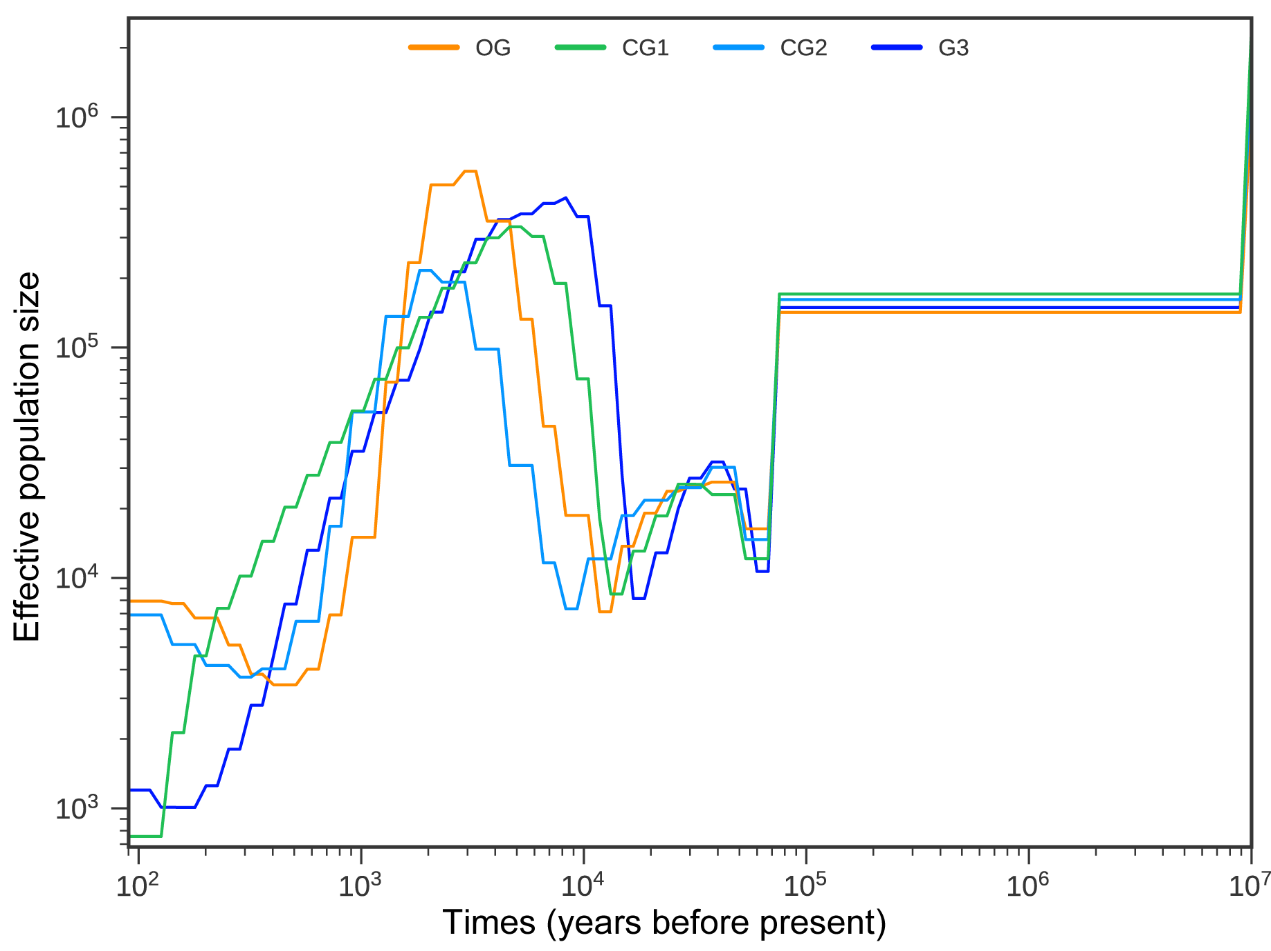


**Fig. S4** Effective population size *Ne* of four garlic populations estimated using the GBS SNPs by the SMC++ method.


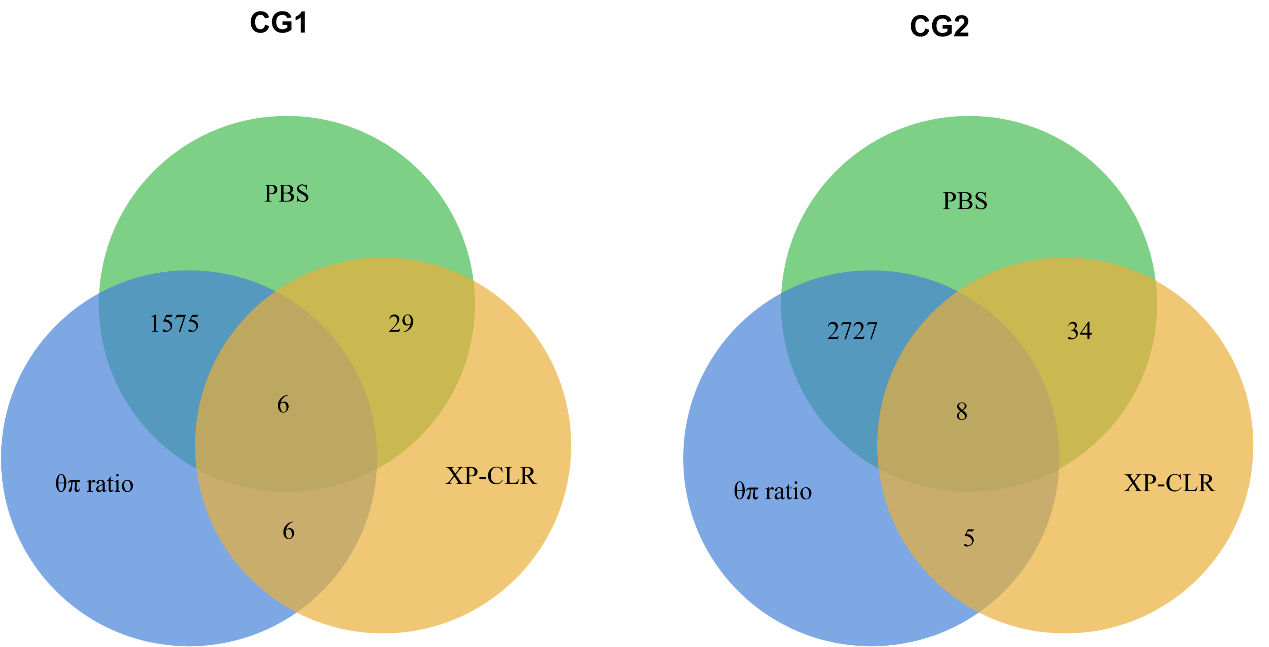


**Fig. S5** A Venn figure shows the selective gene number by the population branch statistic (PBS, green), the θπ ratio (π_OG_/π_CG_, blue), and the test of cross-population composite likelihood ratio (XP-CLR, yellow).


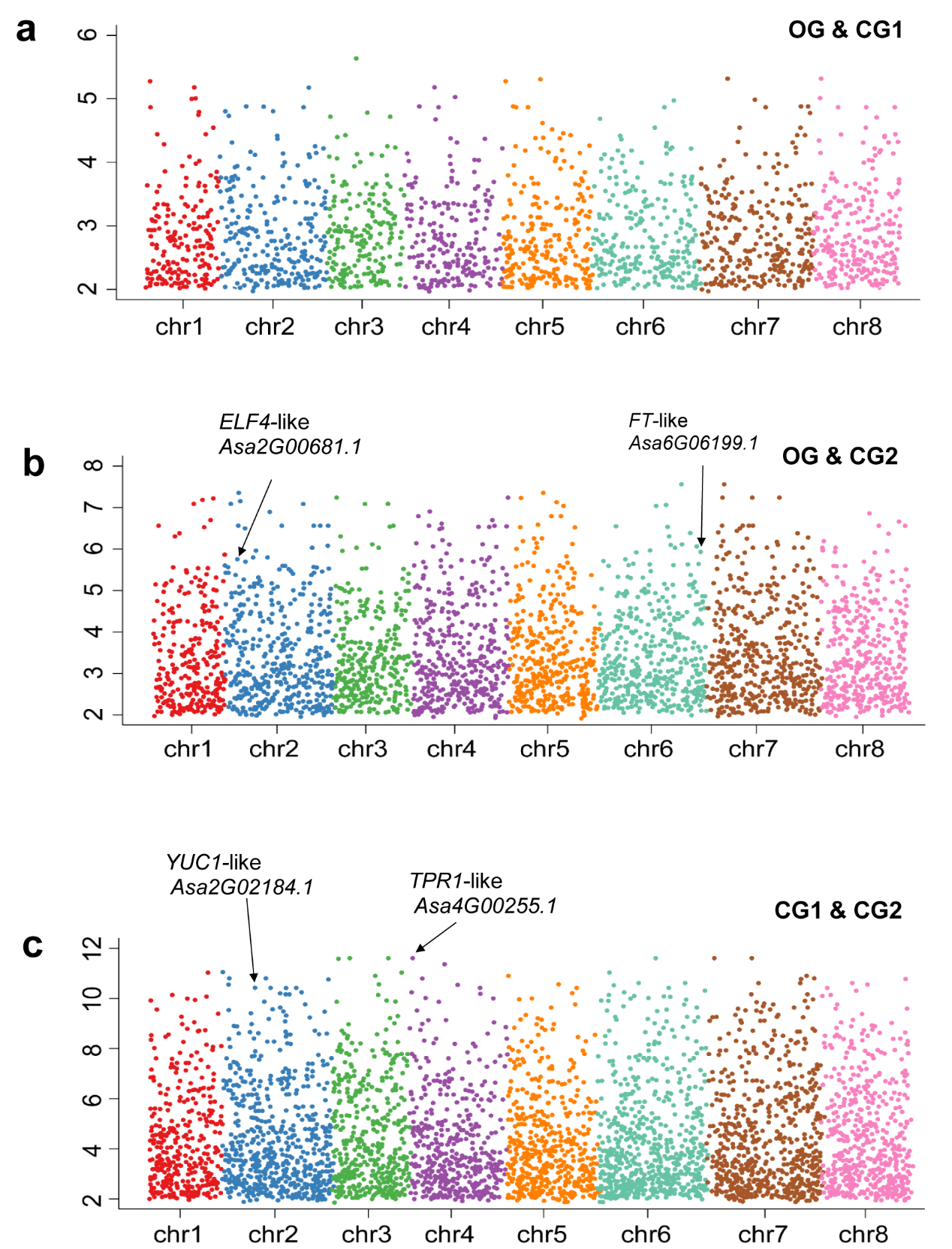


**Fig. S6** Comparison of gene expression level among three groups. Manhattan plot shows the *P* value for the difference of transcript abundance of each gene between two groups by ANOVA analysis. The y axis indicates the -log10(*P* value).


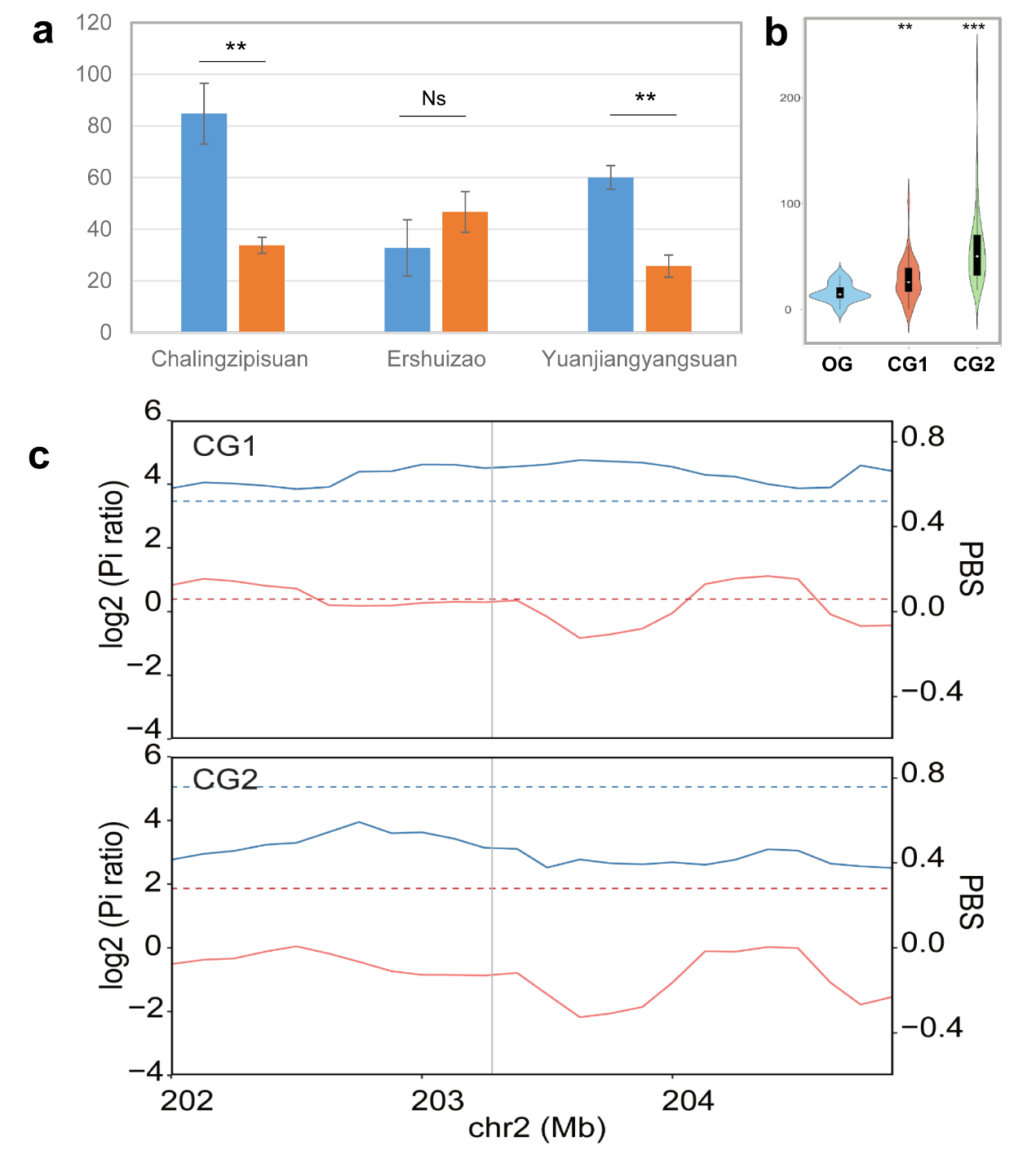


**Fig. S7** Characterization of *ELF4*-like *Asa2G00681.1*. **a**, Expression level of *Asa2G00681.1* in the bulbs under enlarging growth (blue column) and un-enlargement (orange column) in three varieties. **b**, Expression level of *Asa2G00681.1* in the enlarging bulbs of three garlic groups based on the estimation of FPKM value. In the Figure **a** and **b**, the y axis indicates the FPKM value, and Ns indicates no significant difference, and ** and *** indicate a significant difference at the level of 0.01 and 0.001, respectively. **c**, Distribution of the ratio of nucleotide diversity (π_CG_/π_OG_) and *F*_ST_ values in the region of 202.0-204 Mb on chromosome 2. The grey line indicates the position of *Asa2G00681.1*.


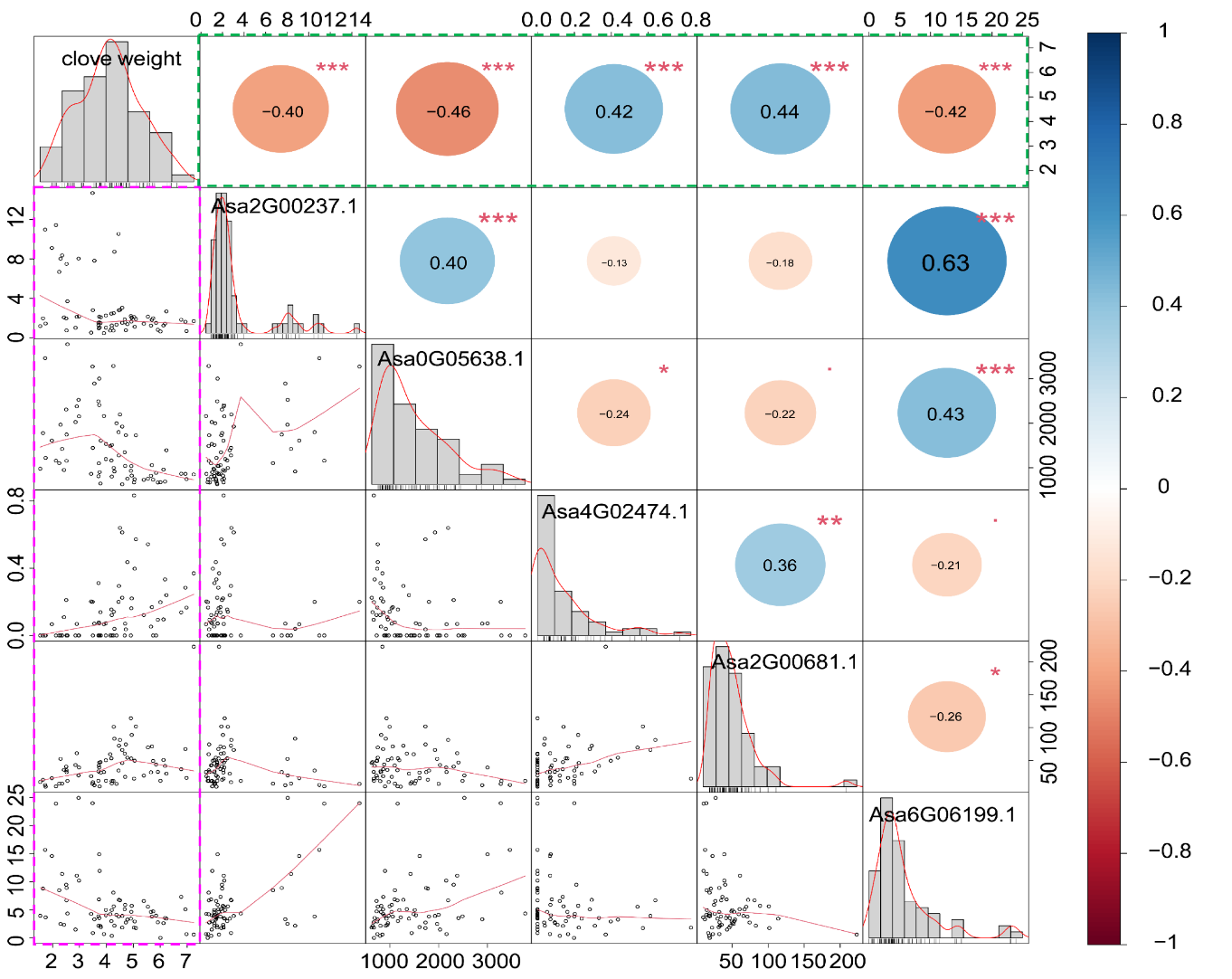


**Fig. S8** Correlation between the transcript abundance of five selective candidates and the clove weight traits in 81 accessions. The number in the top right panes (green dashed box) represents the correlation coefficient, and the red and blue circle indicates the negative and positive correlation, respectively; *** indicated a significance at 0.001 level. The column diagram in the diagonal panes shows the distribution of trait phenotype and expression FPKM value of genes, in which the red line indicates a distribution trend. The scatter plot in the bottom left panes (purple dashed box) shows the correlation between the transcript abundances and the clove weight traits in 81 accessions, where the red line indicates a trend.


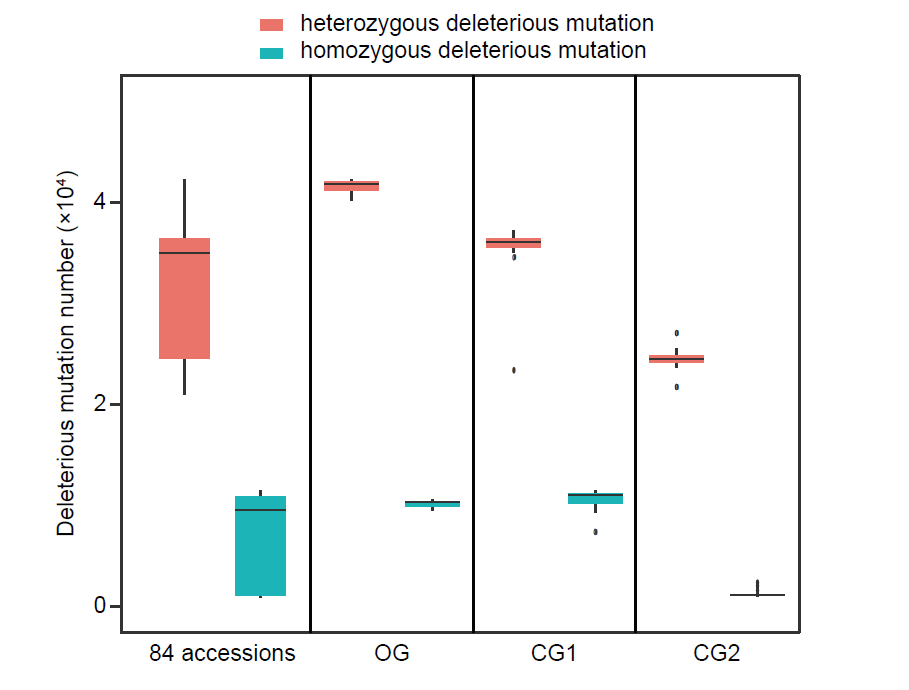


**Fig. S9** Number of heterozygous and homozygous deleterious mutations in 84 garlic accessions and three groups (OG, CG1, CG2). Each box represents the mean and interquartile range.


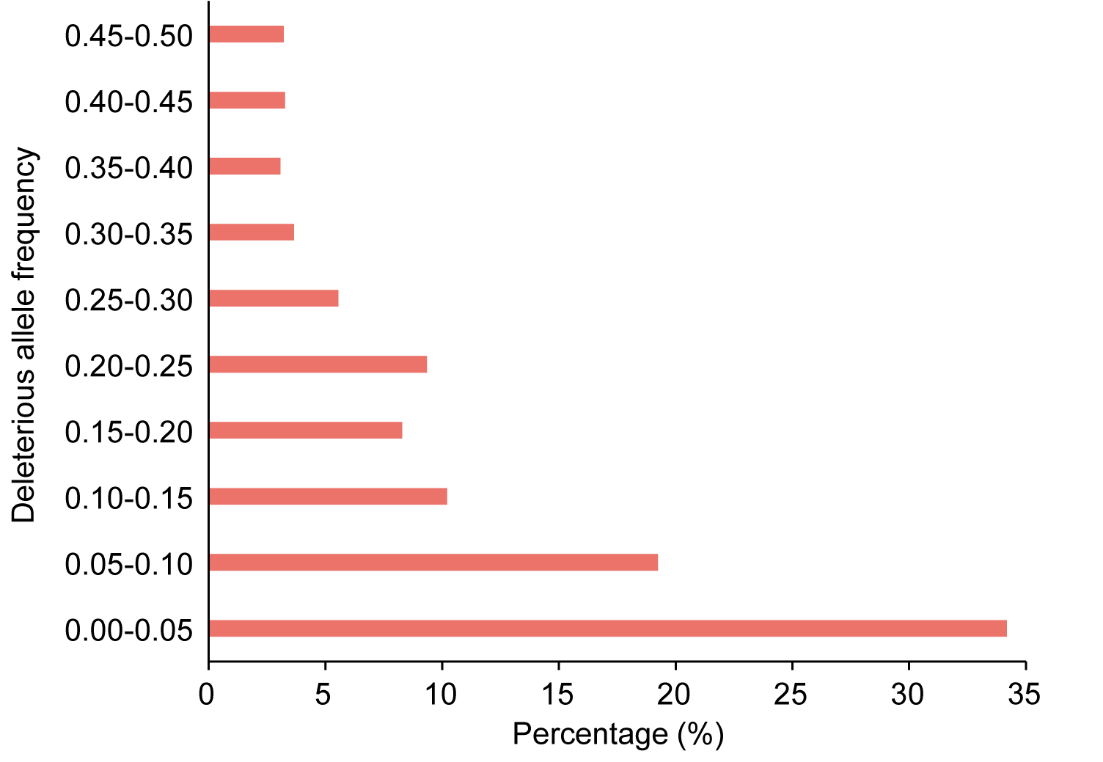


**Fig. S10** Distribution of deleterious allele frequency in 84 garlic accessions


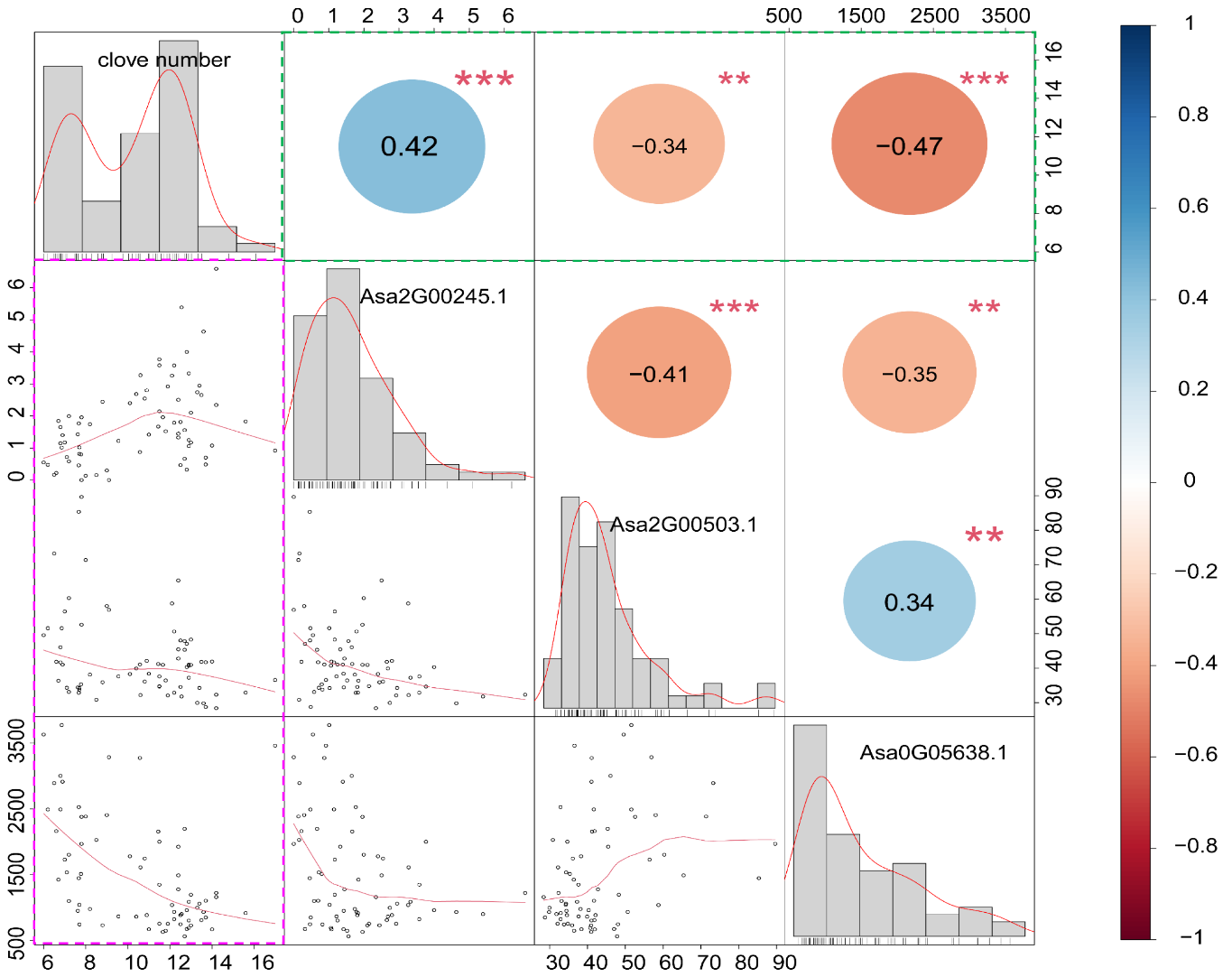


**Fig. S11** Correlation between the transcript abundance of three selective candidates and the clove number traits in 81 accessions. The number in the top right panes (green dashed box) represents the correlation coefficient, and the red and blue circles indicate negative and positive correlation, respectively; ** and *** indicate a significance at 0.01 and 0.001 level. The column diagram in the diagonal panes shows the distribution of trait phenotype and expression FPKM value of genes, in which the red line indicates a distribution trend. The scatter plot in the bottom left panes (purple dashed box) shows the correlation between the transcript abundances and the clove number traits in 81 accessions, where the red line indicates a trend.


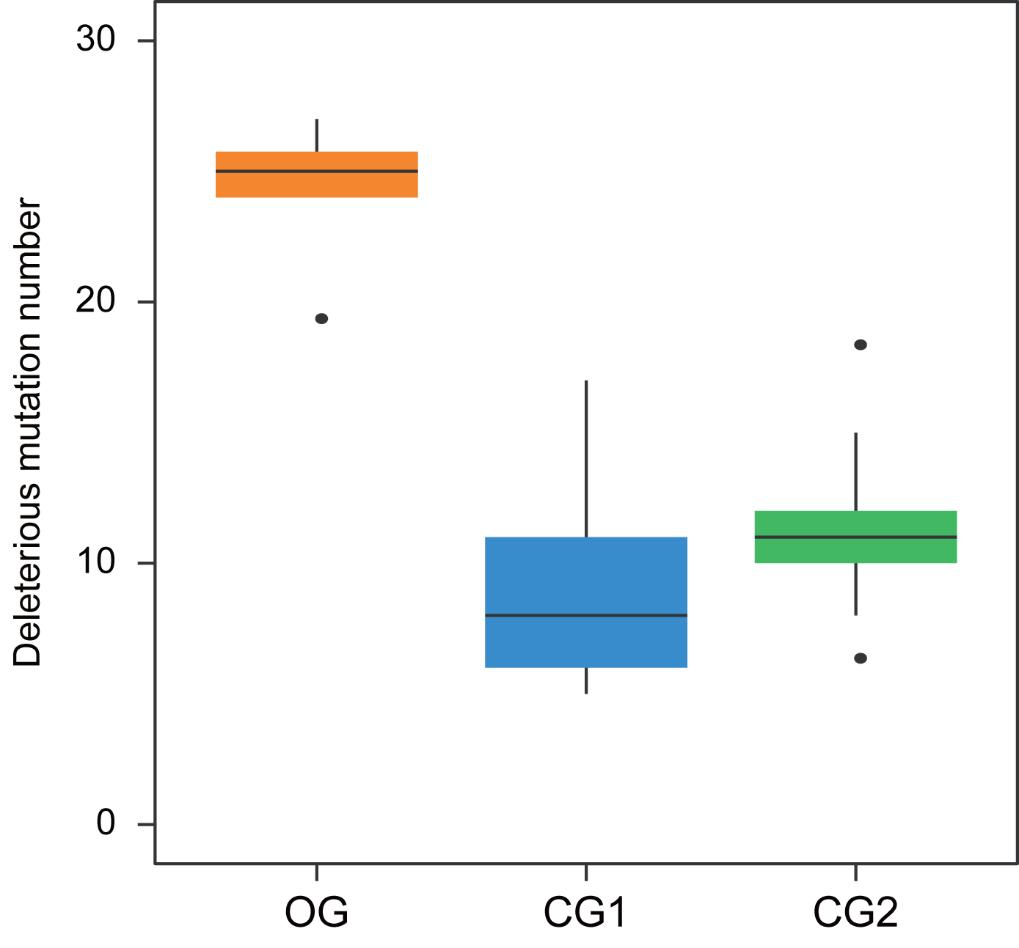


**Fig. S12** Comparison of deleterious mutations burden in the coding region of *Asa2G00245.1* among OG, CG1 and CG2.


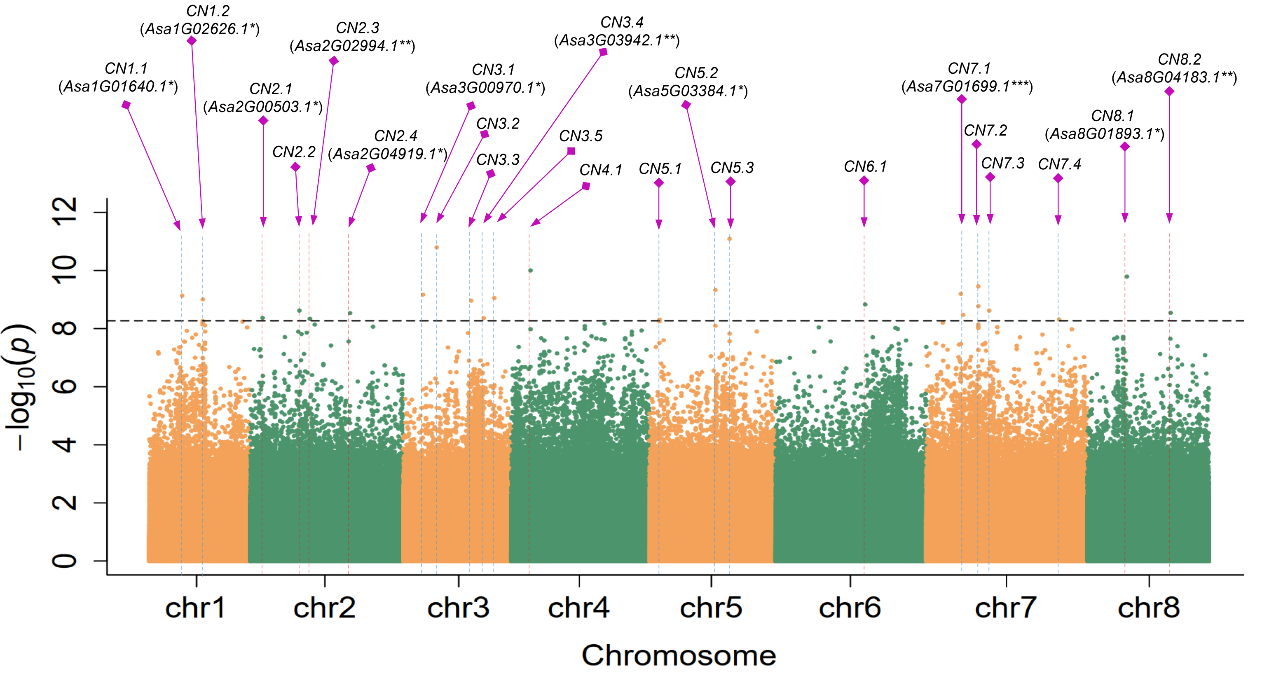


**Fig. S13** Whole genome association analysis of the clove number trait in 230 garlic accessions. Purple arrows indicate the associated position with significant signal (*P* < 5.32 ×10^-9^). The gene under associated loci indicates that it is near to the associated signal (within 200 kb), and its expression shows the significant correlation with the clove number trait in 81 transcriptome-examined accessions, and is considered as the candidate of corresponding loci. *, **, and *** represent the significant association at the level of 0.05, 0.01, and 0.001, respectively.


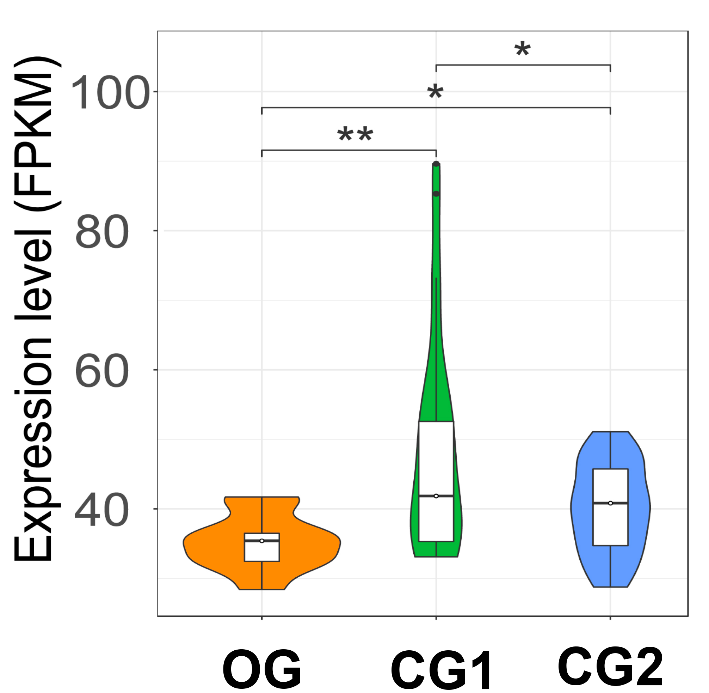


**Fig. S14** Expression level of *Asa2G000503.1* in the enlarging bulbs of three garlic groups based on the estimation of FPKM value. *, and ** indicate the significant difference at the level of 0.05 and 0.01, respectively.


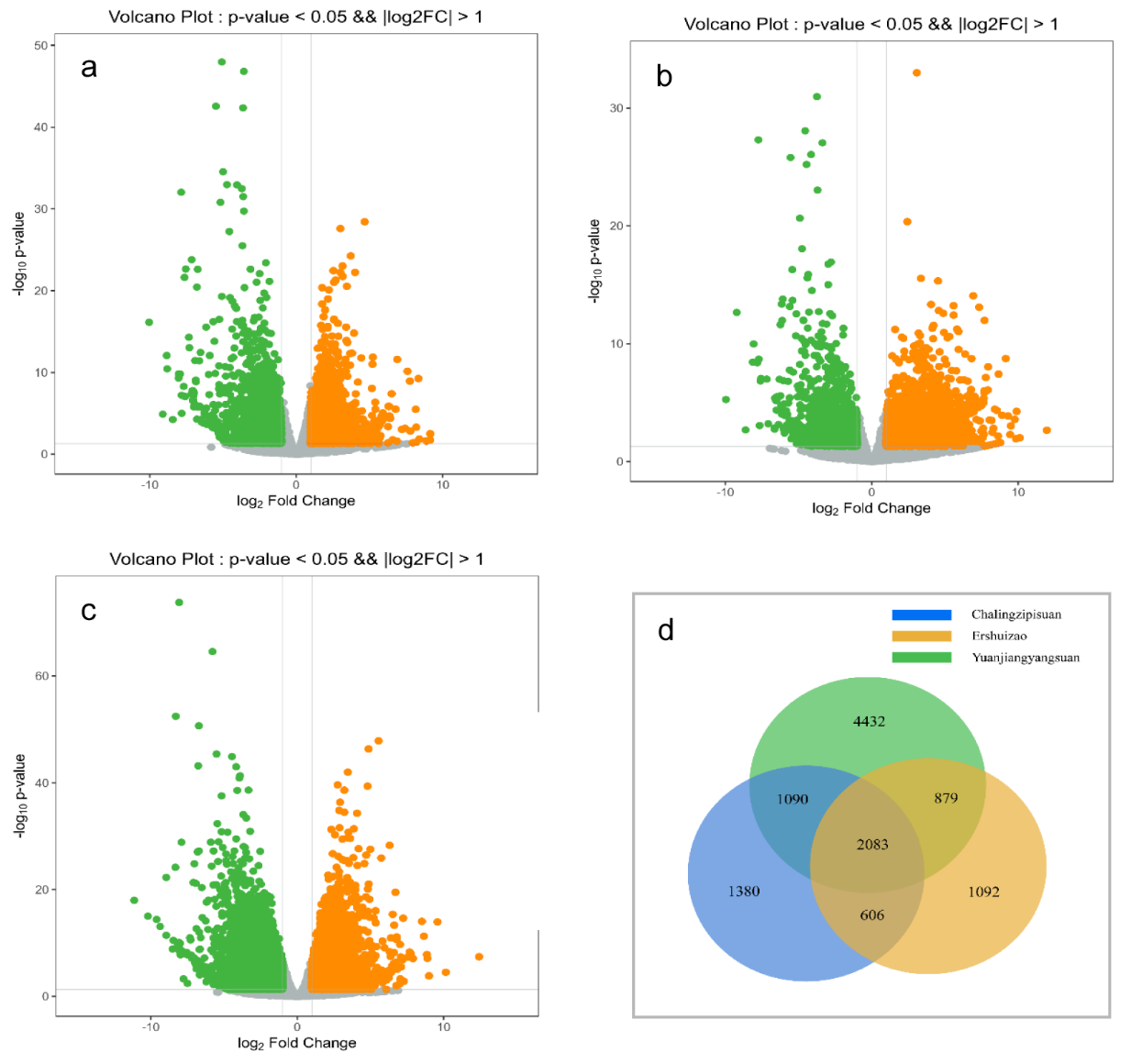


**Fig. S15** Differentially expressed genes (DEGs) in the bulbs whose cloves are before enlargement and under enlarging-growth in three varieties, Ershuizao (**a**), Chalingzipisuan (**b**), and Yuanjiangyangsuan (**c**). Orange dots represent genes with more transcripts in the library of enlarging bulbs, green dots represent genes with fewer transcripts in the library of enlarging bulbs, and grey dots indicate genes whose expression are not changed significantly. **d**, Venn diagram of differentially expressed genes from three varieties.


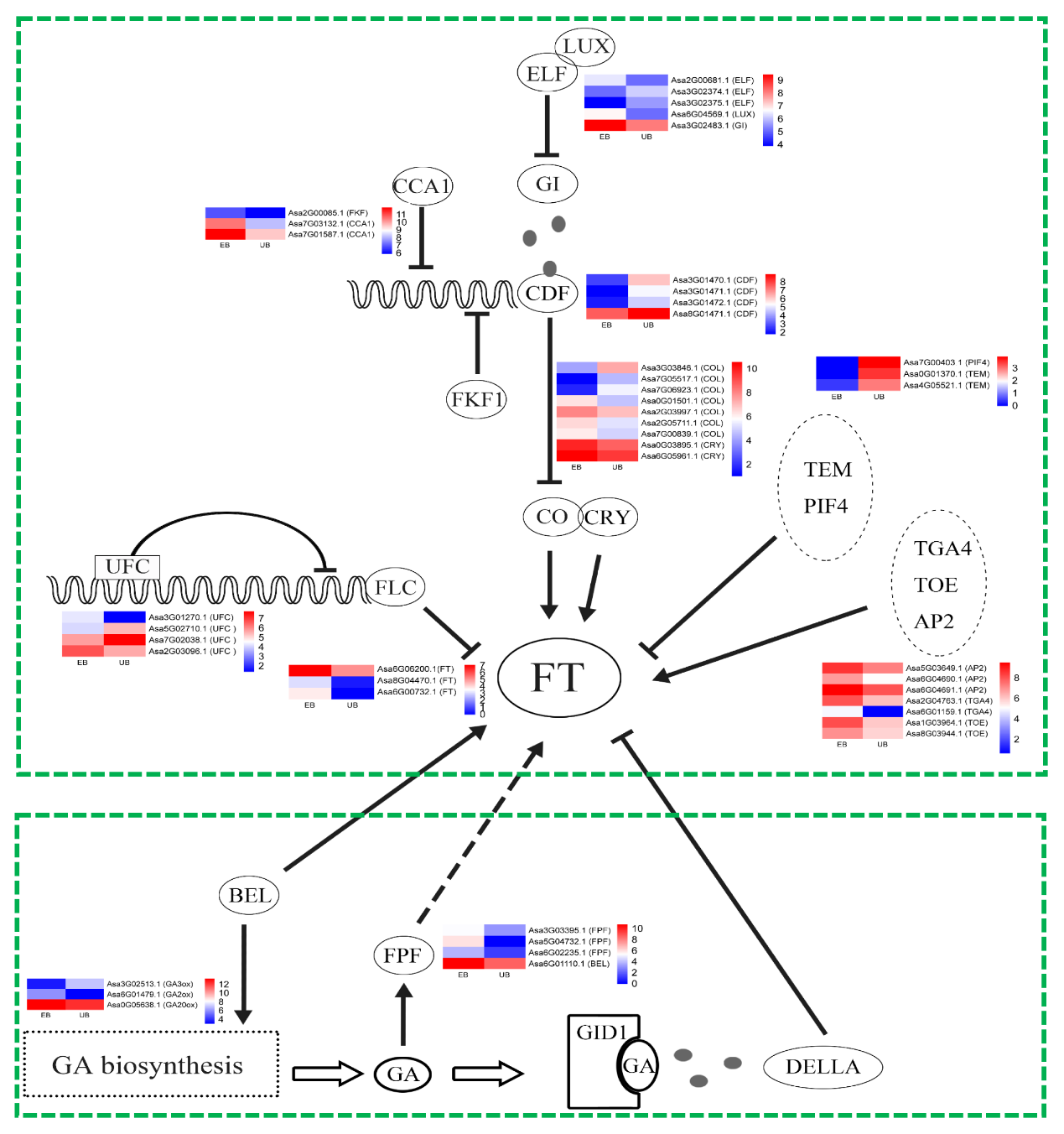


**Fig. S16** A putative model for clove enlarging growth is proposed according to the orthologous analysis, in which 38 and 7 differentially expressed genes are the homologous genes involved in the photoperiod and gibberellin signal pathway. Heatmap shows the expressed difference of candidates between the bulbs whose cloves are under enlarging-growth (EB) and un-enlargement (UB).


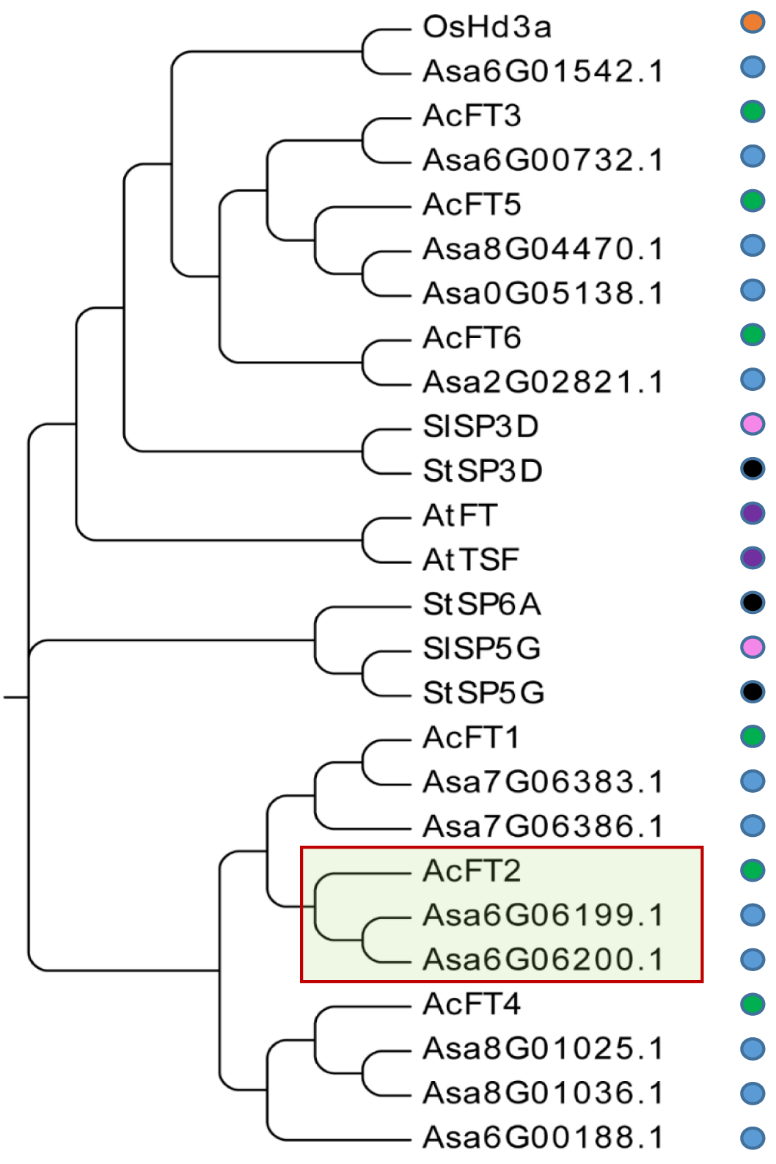


**Fig. S17** Phylogenetic tree of FT and FT-like proteins from six species, including garlic (blue), onion (green), Arabidopsis (purple), rice (orange), potato (black), and tomato (pink).


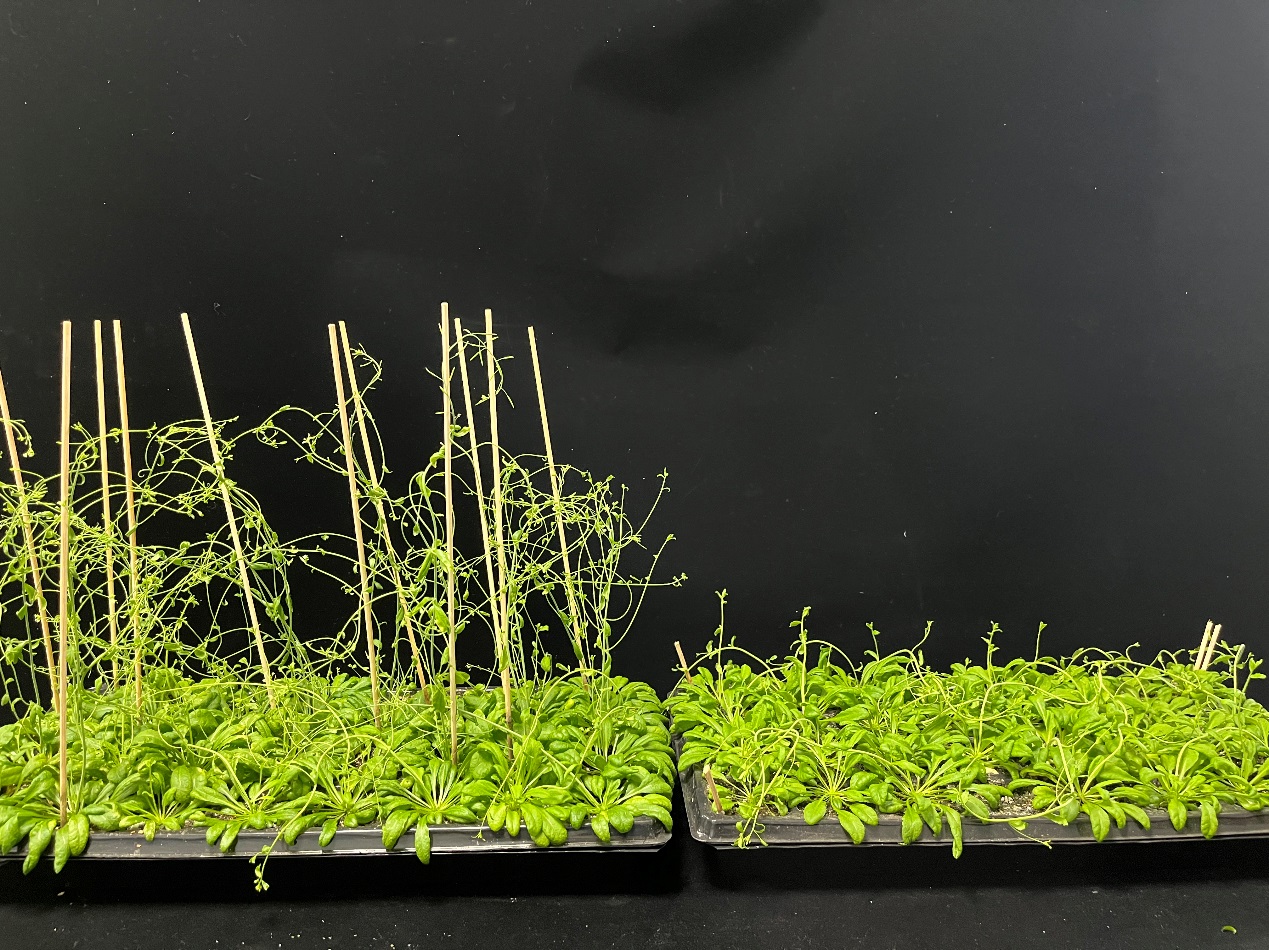


**Fig. S18** Comparison of the flowering time between wild (right) and *Asa06G06199.1*-overexpressed Arabidopsis (left).


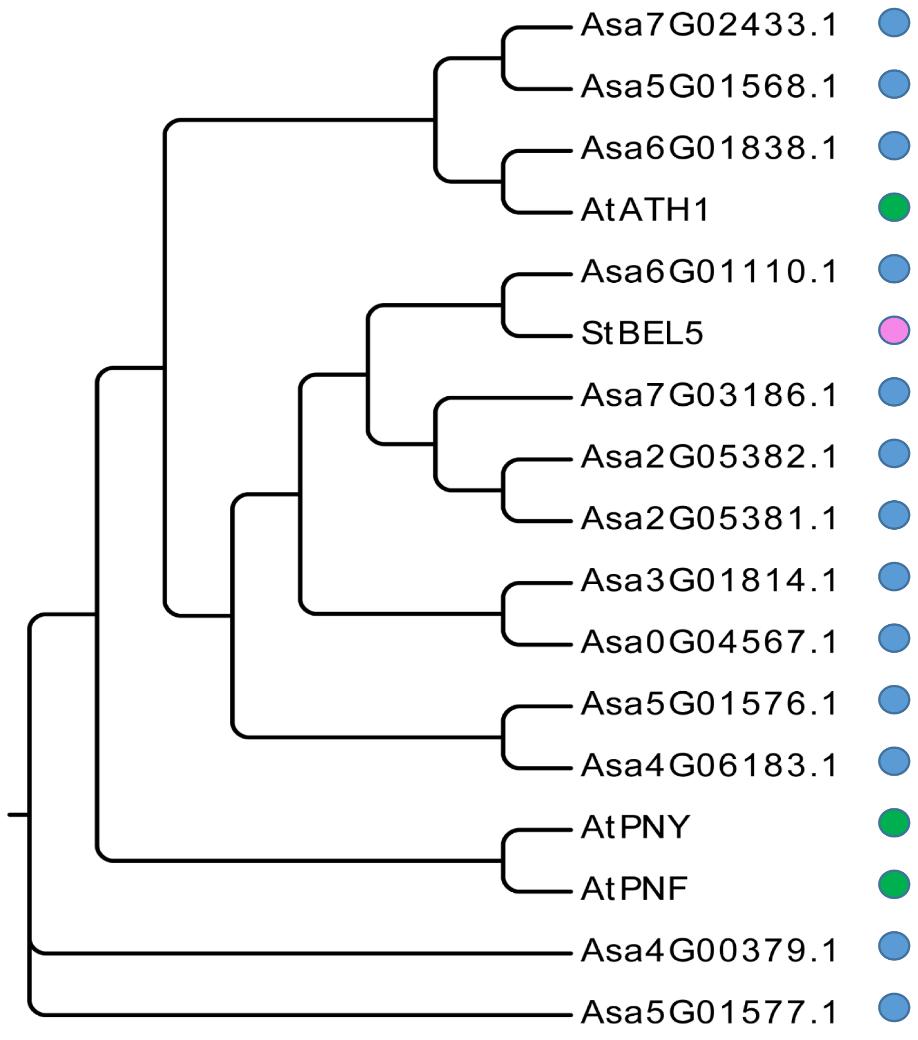


**Fig. S19** Phylogenetic relation of garlic BEL1-like Homeodomain (BELL) members and Arabidopsis and tomato homologs, AtATH1, AtPNY, AtPNF, and StBEL5. The proteins of garlic, Arabidopsis and tomato are indicated by blue, green, and pink circle.


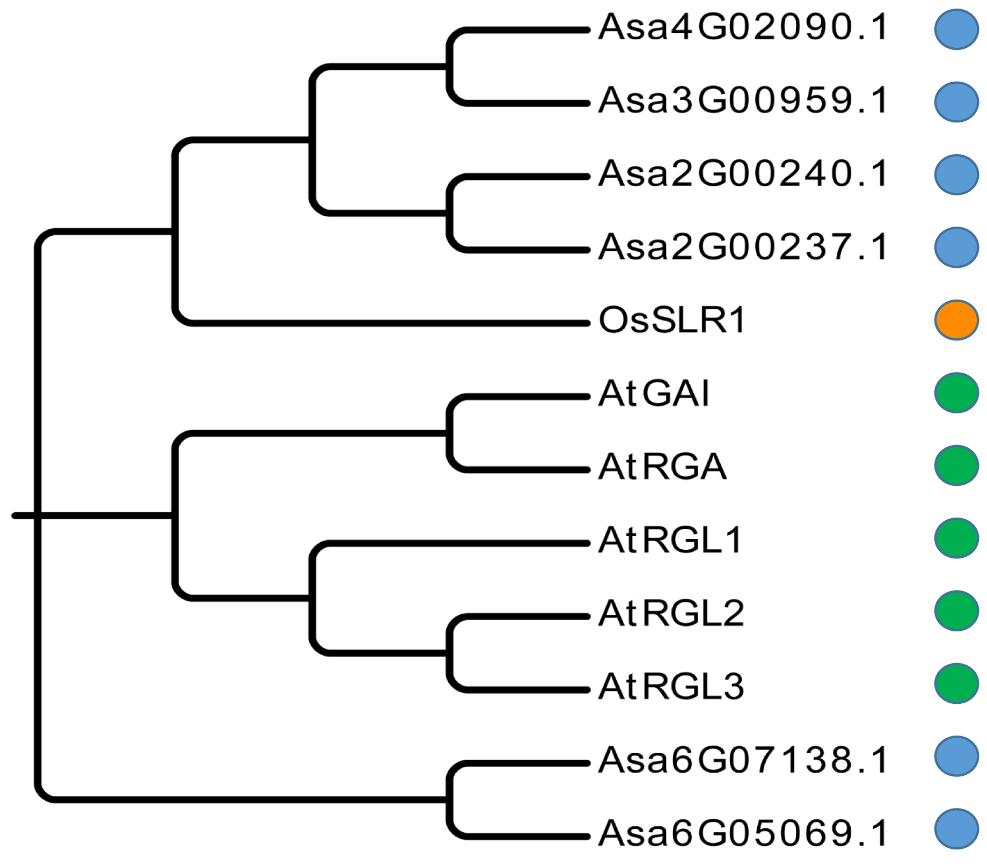


**Fig. S20** Phylogenetic tree of DELLA proteins from three species, including garlic (blue), Arabidopsis (green), and rice (orange).


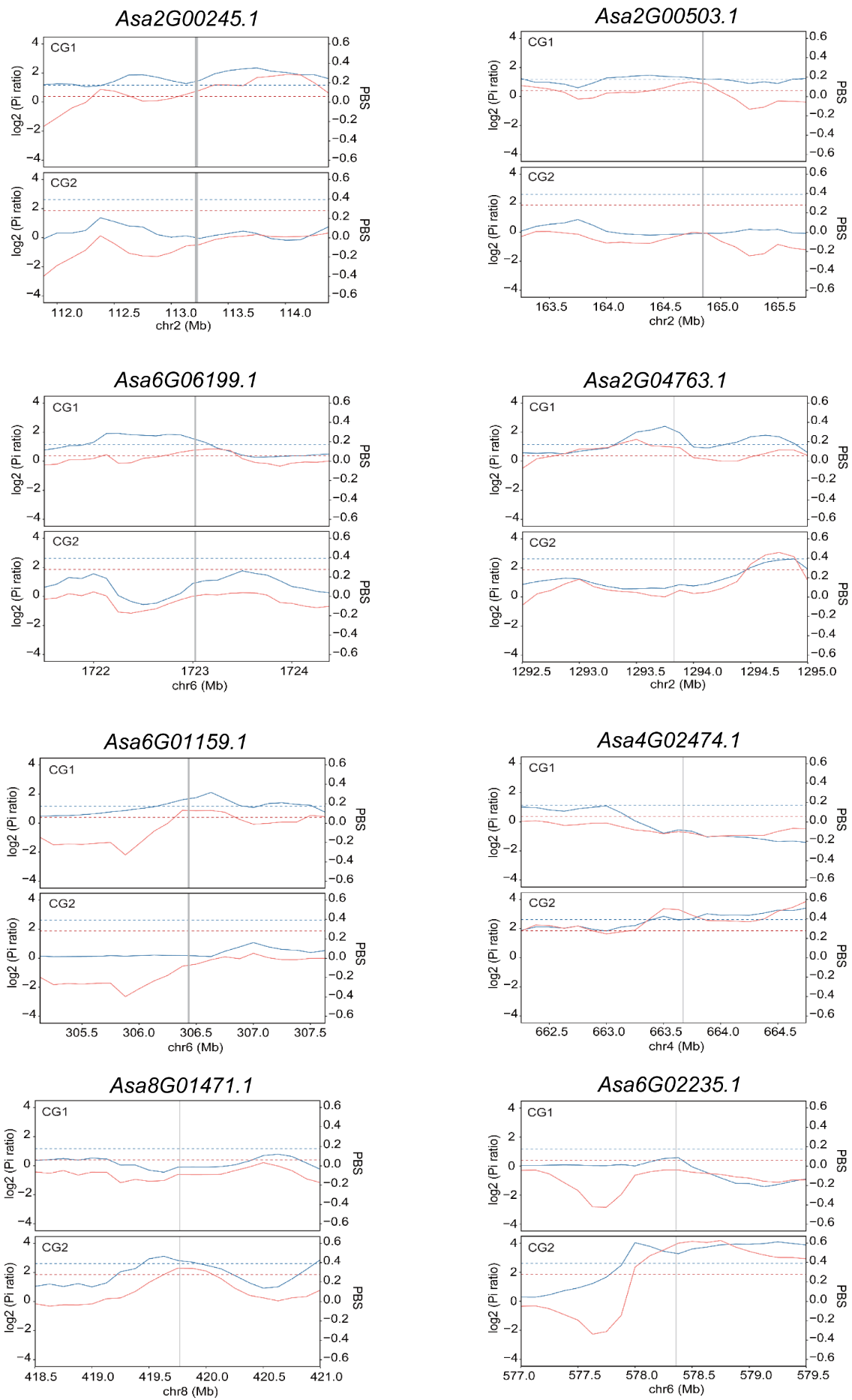


**Fig. S21** Distribution of the ratio of nucleotide diversity (π_CG_/π_OG_) and *F*_ST_ values in the region near to the bulb traits-related candidates, which underwent selection in either CG1 or CG2.
