# Supplementary material for: Genomic insights into the evolutionary history and diversification of bulb traits in garlic
Source: Genome Biol. 2022 Sep 7;23:188. doi: 10.1186/s13059-022-02756-1 (PMC9450234; doi:10.1186/s13059-022-02756-1)
Supplement: Supplementary file 3 — Additional file 3:. Review history. [file 13059_2022_2756_MOESM3_ESM.docx]

Review History

**First round of review**

**Reviewer 1**

**Were you able to assess all statistics in the manuscript, including the appropriateness of statistical tests used?**

There are no statistics in the manuscript.

**Comments to author:**

The manuscript by Li et al explored the population structure of 233 garlic accessions by BGS technology. According to their phylogenetic relationships, the authors selected 84 to generate a genomic variation map. Furthermore, the genetic basis of garlic domestication were investigated using the generated variants, and some candidate genes were proposed. These data will be important resource for the community. However, I have several major concerns about the results.

Specific comments:

1. The authors estimated that OG, CG1 and CG2 diverged about 500,000 years ago. It is well known that human domestication has a history of ~10,000 years, and thus the genetic divergence between CG1 and CG2 should NOT be mainly derived from human selection. To explore the genetic basis of domestication, the direct progenitors of group CG1 and CG2 should be included. In this manuscript, using the accessions of OG as the wild garlics is not suitable for domestication study. In addition, the wild accessions should have higher genetic diversity than cultivated group, in this manuscript its genetic diversity is lower than the majority groups in Fig. 1d, this implies that OG might not be the wild group of garlic.

2. According to the PCA and neighbor joining tree, the authors defined five groups (OG, CG1, CG2, G3 and G4). However, the relationships between PCA and NJ tree are not consistent. From the NJ tree, OG is closer to the three A. ampeloprosum accessions than G3 and G4, but PCA results indicate the opposite relationship.

3. In Fig. 1a the color of dots should be consistent with the color in Fig. 1c to indicate the distribution of the five groups in the garlic population.

4. In page 14 "However, none of the identified candidates underwent common selection in CG1 and CG2". The author should make clear the ratio of candidate genes for clove number and weight undergo common selection in both CG1 and CG2, rather than list only 8 genes in supplementary Fig. S19.

5. In addition to phylogenetic relationships and principal components analysis, the population structure analysis is also required to clarify the genetic diversity of garlic germplasms.

6. In the manuscript, the functions of genes used as examples such as YUUCA and TPRs are supported by few evidence in garlic or other Allium species. As we know, cloves of garlic are abnormal buds, and the formation and development of cloves maybe complex and different from that in model plants. The different expression and correlation analysis can't provide solid evidences for gene functions.

7. In Page 9, "Notably, among these genes underwent expression evolution, 97.4% and 95.6% in CG1 and CG2 were in the outside of selective genomic region". Why?

8. Wrong number of Supplementary table in excel file. Table S16 should be Table S17.

**Reviewer 2**

**Were you able to assess all statistics in the manuscript, including the appropriateness of statistical tests used?**

No, I do not feel adequately qualified to assess the statistics.

**Comments to author:**

This study mainly surveyed the genetic variation and expression diversity among garlic species (Allium sativum) through genome resequencing and RNA-seq technologies. And they further divided this species into three subpopulations and investigated trait-associated genetic variants and gene expression differences. Based on these, the author proposed independent domestication events in two cultivated groups and identified some trait-related genes.

Garlic is cultivated and consumed worldwide, but its genetic study has been postponed by the lack of a reference-grade genome. The recently published garlic reference genome facilitated this field, but there are some caveats that we should be aware of when we are applying and explaining sequencing-based studies. Regarding to the analyses applied and inferences concluded in this study, I have the following concerns: (the line number doesn't match the real ones, but I use them to locate the sentences.)

(1) Many association analyses have been done in this study, but no phenotypic data is available, which makes the readers impossible to check on the results.

(2) In the population structure analysis:

(2.1) The numbers of exonic variants in supplementary table S1 don't match. The total mutant number of the four categories is 8369, while the number of exonic class is 8181. The author should double check to confirm there are no systematic errors in their calculations through the whole study.

(2.2) Page 5 line 54-60: There isn't causal relationship between the read mapping ratio and the genome collinearity, so this collinearity is not supported. For example, large inversions or translocations won't decrease the mapping ratio.

(2.3) In the PCA plots (Fig. 1b), the G3 and G4 populations are both close to the outgroup and the OG (origin group) is the furthest to any of the rest, indicating the longest genetic distance form OG to the others. In common sense, the outgroup (OUT) should be the furthest one. And this PCA plot doesn't support five-group division (page 6 line 1-5).

(2.4) The author hasn't pointed out if the tree in Fig. 1c is rooted. However, the OUT accessions are used as outgroup, so the tree should be rooted on this OUT population. Currently the rooting is based on a clade containing both OUT and OG, which conflicts the common sense. And this incorrect rooting leads to the problematic "monophyletic clade" interpretation (page 6 line 7-9).

(2.5) The G3 and G4 populations are in the middle of CG1 and CG2 populations. What are they? If CG1 and CG2 are cultivated groups, are G3 and G4 also cultivated? Or are they wild species? There is no description of them, but this may help support the independent domestication events in CG1 and CG2.

(3) In the "genomic variation map" section, the read mapping ratio averages 99.05% (page 6 line 42), which is much higher than expected, in the consideration of an inter-species alignment. I would guess most of the reads were partially mapped since this is common in BWA-MEM alignments. Then, if that is the case, I don't think this number is appropriate to show the mapping efficiency. It will be better if the author could show the proportion of genome can be covered by at least two reads in each resequencing sample, which measures the genome size covered effectively for variant calling.

(4) In the "population diversity and differentiation" section

(4.1) Page 7 line 15-19: Are there any references supporting this conclusion? I don't think the conservation surrounding restriction enzyme cutting sites is the reason of the lower nucleotide diversity from GBS analysis compared to that from the genome resequencing. I believe it is mainly because of the lower sampling size in GBS, because GBS can only detect variants surrounding these sites. So, the author should adjust their calculation approach to reach a comparable level of π from GBS analysis.

(4.2) Page 7 line 23-25: There are higher nucleotide diversity in origin group (OG) than cultivated groups (CG1 and CG2), which indicates problems in sampling, for example insufficient sample size or lack of randomness. This is critical because most of the downstream analyses will be affected, including selective sweep analysis and Fst estimation.

(4.3) Page 7 line 34-48: Variance in one trait could be due to variation in a few loci, so I don't think the "large differentiation among these three garlic" groups is well supported by this analysis.

(5) In the "independent domestication of cultivated CG1 and CG2" section:

(5.1) Page 8 line 26-29: Are there domestication before the divergence of CG1 and CG2? Why can't CG1 and CG2 be cultivated in similar environments simultaneously even they can't hybrid? Are there any citations for this statement?

(5.2) Page 8 line 41-50: Essential details need to be clarified for the approach applied here. For example, what are the two populations compared in XP-CLR analysis?

(5.3) Page 8 line 60 - page 9 line 2: Is it possible that the low overlapping results from the use of PBS approach? From supplementary figure S3, we can see most genes are contributed by PBS and π approaches, while PBS indicator is used to identify species-specific fixation, which may exclude those sites shared by CG1 and CG2. Besides, the comparison done in XP-CLR analysis is unclear, so it's hard to tell why it has so few overlaps to the other two methods.

(6) In the "evolutionary selection reshaping the transcriptome of CG1 and CG2" section:

(6.1) It is not good to compare gene expression among individuals in garlic, because the RNA-seq read mapping ratio ranges from 32% to 93%, with an average of 61%, which is pretty low and of high variance. This uneven mapping ratio indicates a large mapping bias that can lead to artificial gene differential expression identified among individuals.

(6.2) Page 9 line 23-26: The author should explain the rationale to use Shannon diversity index to measure the "diversity of transcript abundance". Are there any successful cases for this analysis? Or, is this index proved to be effective for gene expression analysis? And most importantly, there is no detail information about how this index is computed for EACH gene among samples. The author should provide their formula and how they choose the individual proportion for each gene, especially for inter-species gene expression comparisons. Then it might be meaningful to compare this index among groups and species (OG versus CG 1 or 2).

(6.3) Page 9 line 32-46: The author should define the uncommon concept "evolved", explaining how they determine if these genes' expression evolved or not. Similarly, the "function roles" requires more explanation, for example, how they define these gene sets or categories, how those LE (lose expression) and EE (emerge expression) are defined, and so on. I believe these concepts are very uncommon in this field. Are there any references?

(6.4) Page 10 line 8-12: Since it is proved in previous results that the differential expression is a result of selection, how do we conclude that the expression change is the driving force of evolution?

(6.5) Page 10 line 13-15: The concepts of "group-specifically expressed" and "intergroup-differentially expressed" require more clarification.

(6.6) Page 10 line 39-36: Is it possible that these genes expression is correlated with the species difference, and the species difference is correlated with the phenotype variance? If yes, these correlations may come from population stratification instead of real association.

(6.7) Page 10 line 48-53: Is it saying that the gene expression evolution caused the expression network changes? Do we need it?

(7) Page 11 line 29-33: Please explain how the homozygous and heterozygous mutations are defined. I failed to get the idea about the relationship of homozygous site number and the evolution of deleterious burden. For example, when comparing a genetic close individual and a genetic distant individual to a reference genome, it is expected that there should be many more homozygous mutations in the distant individual. Does this mean the distant individual have a separate deleterious burden? I think it is normal because the divergence time is different.

**Authors Response**

**Point-by-point responses to the reviewers’ comments:**

Reviewer #1:

The manuscript by Li et al explored the population structure of 233 garlic accessions by BGS technology. According to their phylogenetic relationships, the authors selected 84 to generate a genomic variation map. Furthermore, the genetic basis of garlic domestication were investigated using the generated variants, and some candidate genes were proposed. These data will be important resource for the community. However, I have several major concerns about the results.

*Response: Thanks for your positive evaluation and suggestions.*

1. The authors estimated that OG, CG1 and CG2 diverged about 500,000 years ago. It is well known that human domestication has a history of ~10,000 years, and thus the genetic divergence between CG1 and CG2 should NOT be mainly derived from human selection.

*Response: We agreed you. We have explained these in the second and third paragraph of the “Discussion” section.*

To explore the genetic basis of domestication, the direct progenitors of group CG1 and CG2 should be included. In this manuscript, using the accessions of OG as the wild garlics is not suitable for domestication study. In addition, the wild accessions should have higher genetic diversity than cultivated group, in this manuscript its genetic diversity is lower than the majority groups in Fig. 1d, this implies that OG might not be the wild group of garlic.

*Response: Yes, OG accessions is nearer to wild species, but not wild species. The direct progenitors of cultivated garlic were uncertain, which have been explained in the section of “Introduction”. Therefore, evolutionary analysis using its progenitors still is challenged. Alternatively, we used the accessions collected from the original places as the compared group (OG), and they had a closer genetic relationship with the accessions of outgroup species than the others. In other words, OG accessions have more genetic information of wild progenitor in their genome, and should be qualified for evolutionary study.*

2. According to the PCA and neighbor joining tree, the authors defined five groups (OG, CG1, CG2, G3 and G4). However, the relationships between PCA and NJ tree are not consistent. From the NJ tree, OG is closer to the three A. ampeloprosum accessions than G3 and G4, but PCA results indicate the opposite relationship.

*Response: Thanks for this thoughtful comment. In the revision, we have shown a rooted tree for these 233 accessions. In addition, three principal components were used to group these accessions, and a three-dimensional plot replaced the previous one in Fig 1b. Then, the results are accordance from NJ tree and PCA analysis. Notably, according to the NJ tree and PCA analysis, together with the Bayesian clustering, the G3 and G4 in previous version showed a close genetic relationship, and they were merged as a group in the revision, namely four main garlic groups were identified in this study. This also does not change the conclusion of our article at all. We have corrected the manuscript.*

3. In Fig. 1a the color of dots should be consistent with the color in Fig. 1c to indicate the distribution of the five groups in the garlic population.

*Response: Yes, we have corrected the color of dots in Fig.1a.*

4. In page 14 "However, none of the identified candidates underwent common selection in CG1 and CG2". The author should make clear the ratio of candidate genes for clove number and weight undergo common selection in both CG1 and CG2, rather than list only 8 genes in supplementary Fig. S19.

*Response: Thanks for your suggestions, and we have done it.*

5. In addition to phylogenetic relationships and principal components analysis, the population structure analysis is also required to clarify the genetic diversity of garlic germplasms.

*Response: It is good suggestion. We have added this figure in Fig.1c.*

6. In the manuscript, the functions of genes used as examples such as YUUCA and TPRs are supported by few evidences in garlic or other Allium species. As we know, cloves of garlic are abnormal buds, and the formation and development of cloves maybe complex and different from that in model plants. The different expression and correlation analysis can't provide solid evidences for gene functions.

*Response: You are right. Because of the challenge of genetic studies by sterility and giant genome in garlic, it is difficult to find more evidences for supporting the function of the ortholog of YUUCA and TPRs. Therefore, we have corrected the sentences in manuscript, and indicated that they are potential candidates.*

7. In Page 9, "Notably, among these genes underwent expression evolution, 97.4% and 95.6% in CG1 and CG2 were in the outside of selective genomic region". Why?

*Response: We infer that expression change of these 97.4% and 95.6% genes were caused by their upstream genes that underwent selection, but not by the direct selection in their regulated regions. We have explained it in manuscript.*

8. Wrong number of Supplementary table in excel file. Table S16 should be Table S17.

*Response: I am sorry for our typo error, and we have corrected it.*

Reviewer #2:

This study mainly surveyed the genetic variation and expression diversity among garlic species (Allium sativum) through genome resequencing and RNA-seq technologies. And they further divided this species into three subpopulations and investigated trait-associated genetic variants and gene expression differences. Based on these, the author proposed independent domestication events in two cultivated groups and identified some trait-related genes. Garlic is cultivated and consumed worldwide, but its genetic study has been postponed by the lack of a reference-grade genome. The recently published garlic reference genome facilitated this field, but there are some caveats that we should be aware of when we are applying and explaining sequencing-based studies. Regarding to the analyses applied and inferences concluded in this study, I have the following concerns: (the line number doesn't match the real ones, but I use them to locate the sentences.).

*Response: Thanks for your comment and suggestion.*

(1) Many association analyses have been done in this study, but no phenotypic data is available, which makes the readers impossible to check on the results.

*Response: We have added these phenotype data in supplementary Table S1.*

(2) In the population structure analysis:

(2.1) The numbers of exonic variants in supplementary table S1 don't match. The total mutant number of the four categories is 8369, while the number of exonic class is 8181. The author should double check to confirm there are no systematic errors in their calculations through the whole study.

*Response: I am sorry for our error. We have corrected it. Additionally, we also checked all data of this study, to make certain that there are not any other errors.*

(2.2) Page 5 line 54-60: There isn't causal relationship between the read mapping ratio and the genome collinearity, so this collinearity is not supported. For example, large inversions or translocations won't decrease the mapping ratio.

*Response: Thanks for your suggestion, and we have corrected it.*

(2.3) In the PCA plots (Fig. 1b), the G3 and G4 populations are both close to the outgroup and the OG (origin group) is the furthest to any of the rest, indicating the longest genetic distance form OG to the others. In common sense, the outgroup (OUT) should be the furthest one. And this PCA plot doesn't support five-group division (page 6 line 1-5).

*Response: Thanks for your comment. In the revision, three principal components were used to group these accessions, and a three-dimensional plot replaced the previous one in Fig 1b. The result distinctly revealed a farther genetic relationship in OG than in the others. In addition, according to the NJ tree, PCA analysis, and Bayesian clustering, the G3 and G4 in previous version showed a close genetic relationship, and they were merged as a group in the revision, namely four main garlic groups were identified in this study. This also does not change the conclusion of our article at all. We have corrected the manuscript.*

(2.4) The author hasn't pointed out if the tree in Fig. 1c is rooted. However, the OUT accessions are used as outgroup, so the tree should be rooted on this OUT population. Currently the rooting is based on a clade containing both OUT and OG, which conflicts the common sense. And this incorrect rooting leads to the problematic "monophyletic clade" interpretation (page 6 line 7-9).

*Response: Thanks for this thoughtful comment. In the revision, we have shown a NJ tree rooted at the OUT for these 233 accessions. This rooted tree has been shown in Fig 1c.*

(2.5) The G3 and G4 populations are in the middle of CG1 and CG2 populations. What are they? If CG1 and CG2 are cultivated groups, are G3 and G4 also cultivated? Or are they wild species? There is no description of them, but this may help support the independent domestication events in CG1 and CG2.

*Response: Thanks for your suggestion. All these accessions were the cultivars collected from Canada and Ukraine. We have increased the explanation of these accessions.*

(3) In the "genomic variation map" section, the read mapping ratio averages 99.05% (page 6 line 42), which is much higher than expected, in the consideration of an inter-species alignment. I would guess most of the reads were partially mapped since this is common in BWA-MEM alignments. Then, if that is the case, I don't think this number is appropriate to show the mapping efficiency. It will be better if the author could show the proportion of genome can be covered by at least two reads in each resequencing sample, which measures the genome size covered effectively for variant calling.

*Response: Thanks for this constructive comment. According to the BAM files in which the sixth row CIGAR string show the match length of alignment, we calculated the read ratio with a match length of 100-109 bp, 110-119 bp, 120-129 bp, 130-139 bp, 140-149 bp, and ≥150 bp in 84 accessions, resulting in an average percent of 0.24%, 0.29%, 0.38%, 0.62%, and 2.88%, and 94.83%, respectively (Supplementary Fig S1). Therefore, there were average 99.24% of reads with a match length of ≥ 100 bp in 84 accessions. Actually, all these 84 accessions were the germplasms of a same species, and therefore, it is an intraspecific alignment. We have explained these in the revision, and added a Fig S1 in supplementary material. In addition, according to your advice, we added the proportion of genome can be covered by at least four reads in each resequencing sample in supplementary Table 3.*

(4) In the "population diversity and differentiation" section

(4.1) Page 7 line 15-19: Are there any references supporting this conclusion? I don't think the conservation surrounding restriction enzyme cutting sites is the reason of the lower nucleotide diversity from GBS analysis compared to that from the genome resequencing. I believe it is mainly because of the lower sampling size in GBS, because GBS can only detect variants surrounding these sites. So, the author should adjust their calculation approach to reach a comparable level of π from GBS analysis.

*Response: Yes, you are right. Your view was validated by the following analysis. SNPs from WGS in 84 accessions were selected randomly, and make its number is same with GBS SNPs, and then calculated the π value. Ten repeats were performed, and their π value were listed in Table R1. The results indicated the π value from this analysis was similar to that from GBS SNP. Therefore, we removed this sentence about lower nucleotide diversity resulted from conservation surrounding restriction enzyme cutting sites.*

*Table R1. Based on the WGS-SNP set, 10 random samplings with the same number of GBS-SNPs were performed for nucleotide diversity analysis*

*repeat 1 2 3 4 5 6 7 8 9 10*

*CG1 3.21E-05 3.21E-05 3.21E-05 3.21E-05 3.21E-05 3.21E-05 3.21E-05 3.21E-05 3.21E-05 3.21E-05*

*CG2 2.35E-05 2.35E-05 2.35E-05 2.35E-05 2.35E-05 2.35E-05 2.35E-05 2.36E-05 2.35E-05 2.35E-05*

*OG 2.20E-05 2.19E-05 2.19E-05 2.19E-05 2.19E-05 2.19E-05 2.19E-05 2.20E-05 2.19E-05 2.19E-05*

*Additionally, nucleotide diversity was counted based on 500-kb sliding windows, using genome-wide SNPs. However, because of a low saturation of GBS SNPs in genome, a windowless model was applied to assess genome-wide nucleotide diversity using these GBS SNPs, thereby to avoid the inaccuracy of nucleotide diversity that is caused by many windows with few SNPs. We have corrected these in the revision and explained the calculated difference in the section of “Methods.”*

(4.2) Page 7 line 23-25: There are higher nucleotide diversity in origin group (OG) than cultivated groups (CG1 and CG2), which indicates problems in sampling, for example insufficient sample size or lack of randomness. This is critical because most of the downstream analyses will be affected, including selective sweep analysis and Fst estimation.

*Response: Thanks for this thoughtful comment. The nucleotide diversity in OG is slightly smaller than in CG2 (~6.8%), and ~25.7% smaller than in CG2. Ten accessions of OG were collected from five different countries, representing a widely geographical distribution of them, which indicated the randomness in sampling for OG should be well.*

To estimate the influence of sample size on downstream analyses, we performed an analysis of π and FST value in each group with same sample size. Ten samples were randomly selected in CG1 and CG2, respectively, and were used for estimating the π and FST value. One hundred replicates were performed. Our results revealed that average π value of 100 times replicates using the random ten sample has an accordant trend of change in three groups with those estimated from whole sample, namely, πOG < πCG2 < πCG1 (Fig. R1). Additionally, the average FST values from 100 times replicates using the random ten samples among three groups were similar to those estimated from whole sample (Fig. R2). Therefore, our results indicated that sample size have not significant influence on the downstream π, FST, and selective analysis [log2(πA /πB)].

*Fig. R1 Nucleotide diversity by random sampling of 10 samples for each population with 100 times based on the WGS-SNP set.*

*Fig. R2 FST Values were calculated by random sampling of 10 samples for each population with 100 times based on the WGS-SNP set.*

(4.3) Page 7 line 34-48: Variance in one trait could be due to variation in a few loci, so I don't think the "large differentiation among these three garlic" groups is well supported by this analysis.

*Response: Thanks for this thoughtful comment. According to your suggestion, we have deleted this sentence.*

(5) In the "independent domestication of cultivated CG1 and CG2" section:

(5.1) Page 8 line 26-29: Are there domestication before the divergence of CG1 and CG2?

*Response: Human only have a crop domesticated history of about ten thousand, while our genomic results indicated that CG1 and CG2 diverge since hundreds of thousands of years. Therefore, we believed that there is not domestication before the divergence of CG1 and CG2.*

Why can't CG1 and CG2 be cultivated in similar environments simultaneously even they can't hybrid? Are there any citations for this statement?

*Response: Garlic is sterile, and it propagated by vegetative cloves, which lead to a reproductive isolation among the garlic individuals. Most accessions of CG1 and CG2 were collected from China, and they can be cultivated in similar environments simultaneously. Their reproductive isolation was caused by absolute sterility, but not geographic isolation. We have corrected the sentence, and made the explanation to be clearer.*

(5.2) Page 8 line 41-50: Essential details need to be clarified for the approach applied here. For example, what are the two populations compared in XP-CLR analysis?

*Response: The comparison is CG versus OG, and we have indicated it in the revision.*

(5.3) Page 8 line 60 - page 9 line 2: Is it possible that the low overlapping results from the use of PBS approach? From supplementary figure S3, we can see most genes are contributed by PBS and π approaches, while PBS indicator is used to identify species-specific fixation, which may exclude those sites shared by CG1 and CG2. Besides, the comparison done in XP-CLR analysis is unclear, so it's hard to tell why it has so few overlaps to the other two methods.

*Response: Thanks for this thoughtful comment. This study using three approaches to detect selective sweeps. Of these methods, the genetic diversity π is a classic statistic to detect signals of selection (especially hard sweeps) by assuming that selected regions show a reduced genetic diversity. PBS method can be viewed as a model-based extension of FST, which is very powerful in detecting incomplete selective sweeps over short divergence times; while the XP-CLR method is able to detect ancient selective events. Because three approaches are compatible and complementary, there were more selective regions detected theoretically. However, as the shown in Table R2, the regions underwent common selection from the θπ ratio, PBS, and XP-CLR is only 143.0 Mb (0.88%), 78.0 Mb (0.48%), and 1.6 Mb (0.0098%), respectively. All three approaches indicated a low ratio of genome underwent common selection in CG1 and CG2. Therefore, we believe that low overlapping does not result from the PBS approach, but the independent evolution of two subpopulation.*

*Notably, there was extremely few overlapping of selective region detected by XP-CLR. XP-CLR is a power tool in detecting ancient selective events. Because garlic is sterile and only can reproduces asexually, the ancient selective events were fixed in CG1 and CG2. Moreover, ancient selective events generally result from natural selection. Unlike domesticated selection that frequently focus on some important regions associated with agronomic traits by human and that results in many common selective regions in different subpopulation of a species, natural selection shows strong randomness, thereby to cause less common selective regions than domesticated selection in different subpopulations. Probably, this is a potential reason for low overlapping of selective region detected by XP-CLR.*

*We have explained these in the section of “methods”, and discussed it in the section of “Discussion”.*

*Table R2. Shared genomic region selected in CG1 and CG2 using different methods*

*Methods Selection commonly region between CG1 and CG2 (Mb)*

*θπ ratio 143.0*

*PBS 78.0*

*XP-CLR 1.6*

(6) In the "evolutionary selection reshaping the transcriptome of CG1 and CG2" section:

(6.1) It is not good to compare gene expression among individuals in garlic, because the RNA-seq read mapping ratio ranges from 32% to 93%, with an average of 61%, which is pretty low and of high variance. This uneven mapping ratio indicates a large mapping bias that can lead to artificial gene differential expression identified among individuals.

*Response: As a vegetative propagated crop, garlic accumulates numerous viruses, and many reads from RNA-sequencing derived from the transcripts of viruses. According to the accumulated level of viruses, the mapping ratio varied across accessions. In this study, to avoid some sample with insufficient data to quantify the expression of garlic genes, we examine the number of reads mapped into garlic genome, and accessions with less than 30 million genome-mapped reads were performed for RNA-sequencing once again, thereby to insure all accessions with more than 30 million genome-mapped reads. In previous study, Shemesh-Mayer et al. quantified the expression level of garlic gene using 17.8-21.0 million clean reads for each sample (Shemesh-Mayer et al. 2015, Front. Plant Sci., 6:271), and Yang et al. performed the analysis of expression profiling for four garlic samples using 29.0-34.8 million clean reads (Yang et al. 2021, BMC Plant Biology, 21:174). Therefore, we believe that our data size of each accessions is enough for quantifying the expression level of genes. We have explained these in the manuscript.*

(6.2) Page 9 line 23-26: The author should explain the rationale to use Shannon diversity index to measure the "diversity of transcript abundance". Are there any successful cases for this analysis? Or, is this index proved to be effective for gene expression analysis?

*Response: Shannon index has been proposed to estimate the diversity of transcript abundance by Martınez and Reyes-Valde (Defining diversity, specialization, and gene specificity in transcriptomes through information theory. 2008, PNAS, 105: 9709–9714). We have added the citation for this method article.*

And most importantly, there is no detail information about how this index is computed for each gene among samples. The author should provide their formula and how they choose the individual proportion for each gene, especially for inter-species gene expression comparisons. Then it might be meaningful to compare this index among groups and species (OG versus CG 1 or 2).

*Response: Thanks for your suggestion. The detailed method has been presented in the section of “Methods”.*

(6.3) Page 9 line 32-46: The author should define the uncommon concept "evolved", explaining how they determine if these genes' expression evolved or not.

*Response: Although evolutionary change in gene expression was frequently focused on (such as the 19th-21st citation in the reference list of this manuscript), we are not sure whether our results match the concept of evolution in gene expression. To explain the results scrupulously, we corrected the sentences.*

Similarly, the "function roles" requires more explanation, for example, how they define these gene sets or categories,

*Response: Thanks for your suggestion, and we have added the explanation in the section of “Method” in detail. Additionally, we increased supplementary Table S13 to show correlated results.*

how those LE (lose expression) and EE (emerge expression) are defined, and so on. I believe these concepts are very uncommon in this field. Are there any references?

*Response: Thanks for your comment. We have defined them in the section of “Methods”. To make the result to be exact, we make a strict threshold, namely, we defined genes that have not any expression in all accessions of a group to be considered as an unexpressed gene in this group, whereas gene that expresses in 90% of accessions of a group to be considered as expressed gene in this group. Accordingly, the gene number identified in revision has slight decrease, relative to previous version.*

(6.4) Page 10 line 8-12: Since it is proved in previous results that the differential expression is a result of selection, how do we conclude that the expression change is the driving force of evolution?

*Response: Yes, it is a conclusion without rigor, and we have deleted this sentence.*

(6.5) Page 10 line 13-15: The concepts of "group-specifically expressed" and "intergroup-differentially expressed" require more clarification.

*Response: Yes, we have corrected the sentence to make the explanation to be clearer.*

(6.6) Page 10 line 39-36: Is it possible that these genes expression is correlated with the species difference, and the species difference is correlated with the phenotype variance? If yes, these correlations may come from population stratification instead of real association.

*Response: You comment is very good. We have carefully thought it, and discussed it with our team. All we agreed you about that genomic divergency of garlic population can cause the expression difference of some genes; then their expression changes result in phenotype variance. However, we think that the correlations should come from association, according to the following reasons.*

*First, it is known that expression change of one gene could altered trait which is controlled by this gene (such as overexpression, RNAi). Namely, expression change of gene has association with trait variation.*

*Next, this study identified only 15%-17.5% of garlic genes with expressed difference among three groups, and the expression of the remaining 82.5%-85% of genes was not affected by population stratification. Among these remining genes, the correlation between their expression level and their controlled traits should be independent of population grouping.*

*Thirdly, in this study, bulb traits showed distinct diversification in three groups; coincidentally, our focused genes (such as Asa2G02184.1 and Asa4G00255.1) have potential important effect to bulb traits (with a P value of 1.3×10-13 and 1.4×10-15). Therefore, it seems that their gene expression value can be grouped, which shows well match with the population stratification. That is, our observation that correlation is caused by population stratification is mainly derived from that fact that bulb traits have distinct diversification and can be grouped from three subpopulations. In other words, if one trait varies across accessions, but its phenotype has not distinct grouping among subpopulations, we believe the expression level of trait-controlled genes to be not grouped, i.e., the correlation between the trait and its controlled genes is independent of population stratification.*

(6.7) Page 10 line 48-53: Is it saying that the gene expression evolution caused the expression network changes? Do we need it?

*Response: I am sorry for our unclear explanation. We want to make a conclusion, that is, after diverging and the respective evolution, the transcriptomic architecture of CG1 and CG2 displayed distinct difference. We have corrected the sentence, thereby to make the conclusion to be more exact.*

(7) Page 11 line 29-33: Please explain how the homozygous and heterozygous mutations are defined. I failed to get the idea about the relationship of homozygous site number and the evolution of deleterious burden. For example, when comparing a genetic close individual and a genetic distant individual to a reference genome, it is expected that there should be many more homozygous mutations in the distant individual. Does this mean the distant individual have a separate deleterious burden? I think it is normal because the divergence time is different.

*Response: SNPs cause potential amino acid substitutions, and their effect on protein function were scored using the SIFT algorithm; The amino acid substitution is predicted to be deleterious if the score is ≤ 0.05, and corresponding SNP was considered as a deleterious mutation. Of these deleterious mutations, if the genotype in an individual is homozygous and is different from the referenced genome, we defined it to be homozygous deleterious mutation; if the genotype is heterozygous, and one of its allele is different that of referenced genome, we defined it to be heterozygous deleterious mutation. We have added this explanation in the section of “Methods.”*

*As your saying, the genetic distance of individuals has correlation with the evolution of deleterious mutation. However, an exact information about deleterious burden in each individual (such as deleterious mutant number, heterozygous or homozygous, and their effect) is difficult to be estimated by genetic distance. In this study, we used the SIFT that is a classical and universal algorithm to estimate the effect of each nonsynonymous mutant, thereby to obtain an exact data of deleterious burden for each individual. These have been introduced in the the section of “Methods” in detail.*

*Notably, if there were gene flow among individuals from different subpopulations, these deleterious mutations will appear in different individuals simultaneously, including close and distant individual. In other word, the finding of separate accumulation of deleterious burden in different garlic groups further supported that there is no gene flow between them, and they evolution from two independent routes.*

**Second round of review**

**Reviewer 1**

The authors have addressed most of my major concerns. I still have several specific comments.

1. In the revised manuscript, the authors divided the accessions into four main groups (OG, G3, CG1 and CG2) instead of five. According to the results of population structure, phylogenetic tree and PCA, G3 group seems to be another cultivated group. If the accessions from G3 could be included, the evolutionary history of cultivated garlics will be more clear and interesting.

2. In Fig 1d, the estimated nucleotide diversity of OG group based on GBS is significantly smaller than that from whole-genome sequencing. In common sense, the diversity of wild group should be significantly larger than cultivated groups. However, the diversity of OG from GBS is lower than that of cultivated CG1 and CG2. Pls check these results.

**Reviewer 3**

The authors have addressed most of the concerns suggested by Reviewer #2 and the revised manuscript has been much improved. I just have some minor comments.

1.The collected site, clove-weight and clove-number of some germplasms are missing in Supplementary Table S1. Especially, the phenotypic data of three accessions of Allium ampeloprosum are missing. It is better to provide the complete information. The authors also should give an overview of the phenotypic data in the result part.

2.Three accessions of Allium ampeloprosum or Allium porrum, should be consistent in Supplementary Table S1 and text description.

3."remaining the G3 groups" should be "remaining the G3 group".

4.Although the authors delete G4 group in text description, there are still some germplasms classified into G4 in Supplementary Table S1.

5.It is better to discuss the insufficient sample size or lack of randomness may lead to the higher nucleotide diversity in origin group (OG) than cultivated groups (CG1 and CG2).

6."If one gene has not any transcript observed in the libraries of accessions of OG, but expresses in more than 90% of accessions of CG1 or CG2, we defined that it emerges expression during garlic evolutionary history. Conversely, if one gene expresses in more than 90% of OG accessions, but there was not any transcript observed in the libraries of accessions of CG1 or CG2, we defined that it loses expression during evolutionary history." If the threshold for LE (lose expression) and EE (emerge expression) has any references?

7."Of the deleterious mutations, if the genotype in an individual is homozygous and is different from the referenced genome, we define it to be a homozygous deleterious mutation; if the genotype in an individual is heterozygous, and one of its allele is different that of referenced genome, we define it to be a heterozygous deleterious mutation". If the definition for homozygous deleterious mutation and heterozygous deleterious mutation has any references?

**Authors Response**

**Point-by-point responses to the reviewers’ comments:**

Reviewer #1:

The authors have addressed most of my major concerns. I still have several specific comments.

*Response: Thanks for your evaluation and suggestions.*

1. In the revised manuscript, the authors divided the accessions into four main groups (OG, G3, CG1 and CG2) instead of five. According to the results of population structure, phylogenetic tree and PCA, G3 group seems to be another cultivated group. If the accessions from G3 could be included, the evolutionary history of cultivated garlics will be more clear and interesting.

*Response: Thanks for your suggestions. Because none of G3 accessions were performed whole-genome sequencing, we inferred the population demographic history and gene flows of four groups (G3, CG1, CG2 and OG) based on GBS SNPs, using the SMC++ and D-statistic method, respectively. The result of demographic history showed that the four populations experienced a similar demographic bottleneck around 10,000-1,5000 YBP, which is consistent with the estimation of three populations based on WGS data. However, the inference of older historical dynamics (>100,000 years ago) based on GBS SNPs is not robust enough. We have corrected the fig 2b and added a supplementary figure to show these results.*

2. In Fig 1d, the estimated nucleotide diversity of OG group based on GBS is significantly smaller than that from whole-genome sequencing.

*Response: Thanks for your thoughtful comment. This comment has also been inquired by the reviewer #2 in the first-round revision. To find the reason caused this result, we performed an additional analysis in the last-round revision, and revealed that relatively low nucleotide diversity estimated from GBS comparing that from whole-genome sequencing were caused by a low saturation of GBS SNPs in genome.*

In common sense, the diversity of wild group should be significantly larger than cultivated groups. However, the diversity of OG from GBS is lower than that of cultivated CG1 and CG2. Pls check these results.

*Response: We agree you, and the diversity of wild group should be significantly larger than those of cultivated groups, theoretically. However, in this study, OG accessions is nearer to wild species, but not wild species. Additionally, small sample size and/or a potential bad randomness of sample are the potential reason for the slightly low nucleotide diversity in OG. We have discussed these in the section “Discussion.”*

Reviewer #3:

The authors have addressed most of the concerns suggested by Reviewer 2 and the revised manuscript has been much impr.oved. I just have some minor comments.

*Response: Thanks for your positive evaluation and suggestions.*

1.The collected site, clove-weight and clove-number of some germplasms are missing in Supplementary Table S1. Especially, the phenotypic data of three accessions of Allium ampeloprosum are missing. It is better to provide the complete information. The authors also should give an overview of the phenotypic data in the result part.

*Response: Thanks for your suggestion. Most of missing data about the collected site of germplasms have been added by referencing raw record of them. However, because these germplasms were continuously collected in the past three decades, there were still 23 germplasms that cannot found their certain collected sites.*

*We also replenished the information of phenotypic data for two traits, including those of three accessions Allium ampeloprosum. However, because there were eleven accessions that could not adapt the local environmental condition and failed to harvest the bulbs, we have not collected their phenotypic data of bulb traits. We have explained an overview of the phenotypic data in the section of “Results”.*

2.Three accessions of Allium ampeloprosum or Allium porrum, should be consistent in Supplementary Table S1 and text description.

*Response: Thanks for your suggestion. We have corrected it in the supplementary Table 1.*

3.“remaining the G3 groups” should be “remaining the G3 group”.

*Response: I am sorry for our error, and we have corrected it.*

4.Although the authors delete G4 group in text description, there are still some germplasms classified into G4 in Supplementary Table S1.

*Response: I am sorry for our error, and we have corrected it.*

5.It is better to discuss the insufficient sample size or lack of randomness may lead to the higher nucleotide diversity in origin group (OG) than cultivated groups (CG1 and CG2).

*Response: Thanks for your suggestion, and we have discussed it in the section “Discussion.”*

6.“If one gene has not any transcript observed in the libraries of accessions of OG, but expresses in more than 90% of accessions of CG1 or CG2, we defined that it emerges expression during garlic evolutionary history. Conversely, if one gene expresses in more than 90% of OG accessions, but there was not any transcript observed in the libraries of accessions of CG1 or CG2, we defined that it loses expression during evolutionary history.” If the threshold for LE (lose expression) and EE (emerge expression) has any references?

*Response: The threshold is defined by ourselves. The threshold used in this study is strict (i.e., either none or >90%), and the result is reliable.*

7.“Of the deleterious mutations, if the genotype in an individual is homozygous and is different from the referenced genome, we define it to be a homozygous deleterious mutation; if the genotype in an individual is heterozygous, and one of its allele is different that of referenced genome, we define it to be a heterozygous deleterious mutation”. If the definition for homozygous deleterious mutation and heterozygous deleterious mutation has any references?

*Response: Yes, it has references, and we cited one in the references [47. Zhang, C. et al. The genetic basis of inbreeding depression in potato. Nat. Genet. 51, 374-378 (2019)].*

**Third round of review**

**Reviewer 1**

The authors addressed most of my concerns. No further comments.
